# Biomarker discovery in progressive supranuclear palsy from human cerebrospinal fluid

**DOI:** 10.1186/s12014-024-09507-3

**Published:** 2024-09-28

**Authors:** Yura Jang, Sungtaek Oh, Anna J. Hall, Zhen Zhang, Thomas F. Tropea, Alice Chen-Plotkin, Liana S. Rosenthal, Ted M. Dawson, Chan Hyun Na, Alexander Y. Pantelyat

**Affiliations:** 1 Neuroregeneration and Stem Cell Programs, Institute for Cell Engineering, Johns Hopkins University School of Medicine, Baltimore, MD USA; 2 Department of Neurology, Johns Hopkins University School of Medicine, Baltimore, MD USA; 3 Department of Pathology, Johns Hopkins University School of Medicine, Baltimore, MD USA; 4 Department of Neurology, Perelman School of Medicine, Philadelphia, PA USA; 5 Solomon H. Snyder Department of Neuroscience, Johns Hopkins University School of Medicine, Baltimore, MD USA; 6 Department of Pharmacology and Molecular Sciences, Johns Hopkins University School of Medicine, Baltimore, MD USA

**Keywords:** Progressive supranuclear palsy, Cerebrospinal fluid, Biomarkers, Proteomics, Mass spectrometry

## Abstract

**Background:**

Progressive supranuclear palsy (PSP) is a neurodegenerative disorder often misdiagnosed as Parkinson’s Disease (PD) due to shared symptoms. PSP is characterized by the accumulation of tau protein in specific brain regions, leading to loss of balance, gaze impairment, and dementia. Diagnosing PSP is challenging, and there is a significant demand for reliable biomarkers. Existing biomarkers, including tau protein and neurofilament light chain (NfL) levels in cerebrospinal fluid (CSF), show inconsistencies in distinguishing PSP from other neurodegenerative disorders. Therefore, the development of new biomarkers for PSP is imperative.

**Methods:**

We conducted an extensive proteome analysis of CSF samples from 40 PSP patients, 40 PD patients, and 40 healthy controls (HC) using tandem mass tag-based quantification. Mass spectrometry analysis of 120 CSF samples was performed across 13 batches of 11-plex TMT experiments, with data normalization to reduce batch effects. Pathway, interactome, cell-type-specific enrichment, and bootstrap receiver operating characteristic analyses were performed to identify key candidate biomarkers.

**Results:**

We identified a total of 3,653 unique proteins. Our analysis revealed 190, 152, and 247 differentially expressed proteins in comparisons of PSP vs. HC, PSP vs. PD, and PSP vs. both PD and HC, respectively. Gene set enrichment and interactome analysis of the differentially expressed proteins in PSP CSF showed their involvement in cell adhesion, cholesterol metabolism, and glycan biosynthesis. Cell-type enrichment analysis indicated a predominance of neuronally-derived proteins among the differentially expressed proteins. The potential biomarker classification performance demonstrated that ATP6AP2 (reduced in PSP) had the highest AUC (0.922), followed by NEFM, EFEMP2, LAMP2, CHST12, FAT2, B4GALT1, LCAT, CBLN3, FSTL5, ATP6AP1, and GGH.

**Conclusion:**

Biomarker candidate proteins ATP6AP2, NEFM, and CHI3L1 were identified as key differentiators of PSP from the other groups. This study represents the first large-scale use of mass spectrometry-based proteome analysis to identify cerebrospinal fluid (CSF) biomarkers specific to progressive supranuclear palsy (PSP) that can differentiate it from Parkinson’s disease (PD) and healthy controls. Our findings lay a crucial foundation for the development and validation of reliable biomarkers, which will enhance diagnostic accuracy and facilitate early detection of PSP.

**Supplementary Information:**

The online version contains supplementary material available at 10.1186/s12014-024-09507-3.

## Introduction

Progressive supranuclear palsy (PSP) is a neurodegenerative Parkinsonian disorder with an estimated prevalence of about 5 to 6 in every 100,000 people worldwide, typically beginning after the age of 60 [1–3]. The pathological features of PSP are characterized by progressive accumulation of 4-repeat tau, formation of globose neurofibrillary tangles, and neuronal loss in the brainstem, basal ganglia, and cortex [1]. Two of the classic clinical signs of patients with PSP are impairment of vertical gaze and balance loss with backward falls [4]. The most common initial symptom of people with PSP is balance loss [5] and subsequently, PSP patients show changes in their mood and behavior and develop dementia over time [1–3]. It is recognized that PSP can have different clinical presentations, and the current clinical diagnostic criteria include several variants in addition to the most common PSP-Richardson syndrome [6]. 

The clinical evaluation of early-stage and variant forms of PSP is challenging, with limited sensitivity and specificity, making it difficult to distinguish PSP from alternative diagnoses such as Parkinson’s Disease (PD) [6]. In recent years, diagnostic approaches have evolved, exploiting magnetic resonance imaging (MRI) and positron emission tomography (PET) [7]. Currently, there are no established diagnostic biomarkers of PSP, owing to observed discrepancies between clinical manifestations and underlying neuropathological findings. These inconsistencies hinder their utilization in key areas such as early-stage diagnosis, precise pathological characterization, and longitudinal tracking of disease progression [8]. Further research and development are essential for discovering and optimizing PSP biomarkers, given their potential importance in understanding and managing the disease.

Multiple research groups have endeavored to establish an accurate diagnosis of PSP by identifying specific CSF biomarkers [1, 4]. Since the abnormal accumulation of tau proteins within brain cells is considered a potential target for developing therapeutic interventions for PSP, the predominant studies on CSF biomarkers for PSP were focused on tau proteins [9–11]. However, the relationship between total tau, phosphorylated tau, and tau fraction levels in CSF and the disease’s clinical presentation may be complex, and not necessarily distinct from healthy control (HC) groups in certain contexts [2, 12]. 

On the other hand, multiple studies report that neurofilament light chain (NfL) concentrations are 2 to 5 times higher in the CSF of PSP patients compared to HC and PD groups, and similar results were observed in plasma [3–5]. Nevertheless, the diagnostic specificity of NfL for PSP remains inconclusive [3, 4, 13, 14]. To tackle this challenge, in this study, we conducted a mass spectrometry-based proteomics experiment for the identification of additional biomarkers in CSF of PSP patients. We analyzed 120 CSF samples from 40 PSP, 40 PD, and 40 HC individuals. We exploited 11-plex tandem mass tags (TMTs) to analyze 120 samples more accurately. This study represents a comprehensive mass spectrometry-based proteomic analysis of human CSF from PSP patients, aiming to identify PSP biomarkers that distinguish it from PD and HC. The candidate biomarkers discovered in this study—if validated—will pave the way for the development of reliable PSP biomarkers.

## Materials and methods

### Collection of cerebrospinal fluid samples

We employed CSF samples from 40 PSP, 40 PD, and 40 HC individuals well-matched on gender and age. The CSF samples were collected from study volunteers at the University of Pennsylvania using the previously described Parkinson’s Disease Biomarkers Program CSF collection protocol [15] (procedure Manual: https://biosend.org/docs/studies/PDBP/PDBP%20Manual%20of%20Procedures.pdf). Briefly, the CSF samples were collected from study participants in polypropylene vials, spun down at 2000 x g for 10 min at room temperature (18 °C to 25 °C), aliquoted, and stored at ‒80 °C. Samples were shipped on dry ice to Johns Hopkins and stored at ‒80 °C. The sample information is provided in Table 1. This study was approved by the University of Pennsylvania Institutional Review Board. Informed consent was obtained from each participant at study enrollment in accordance with the Declaration of Helsinki.

**Table 1:** Demographic information of the CSF samples used in this study

		Diagnosis			Total (*N* = 120)	Test
		HC (*N* = 40)	PD (*N* = 40)	PSP (*N* = 40)		
Age.Yrs						*P* value: 0.0253 (Kruskal-Wallis rank sum test)
	Mean (95% CI)	67.7 (65.5; 69.9)	64.1 (61.7; 66.5)	68.8 (66.6; 71.0)	66.9 (64.5; 69.2)	
	Median	66	65	69	66.5	
Sex						*P* value: 0.1864 (Pearson’s Chi-squared test)
	Female	27 (67.5%)	19 (47.5%)	24 (60%)	70 (58.3%)	
	Male	13 (32.5%)	21 (52.5%)	16 (40%)	50 (41.7%)	
Race						*P* value: 4.3e-05 (Pearson’s Chi-squared test)
	Black	10 (25%)	1 (2.5%)	0 (0%)	11 (9.2%)	
	White	26 (65%)	39 (97.5%)	39 (97.5%)	104 (86.7%)	
	More than one race	4 (10%)	0 (0%)	0 (0%)	4 (3.3%)	
	American Indian	0 (0%)	0 (0%)	1 (2.5%)	1 (0.8%)	
Education.Yrs						*P* value: 0.0038 (Kruskal-Wallis rank sum test)
	Mean (95% CI)	15.1 (16.5; 13.8)	17.1 (17.7; 16.5)	14.9 (15.8; 14)	15.7 (16.7; 14.7)	
	Median	16	18	14.5	16	

CI: confidence interval

### Sample preparation for the mass spectrometry analysis

We conducted mass spectrometry analysis of 120 CSF samples using 13 batches of 11-plex tandem mass tag (TMT, Thermo Scientific) experiments. For the normalization of data from 13 batches of the TMT experiment, we included the master pool (MP) in the last channel of each batch. We also included quality control (QC) in 10 batches to monitor data quality. To minimize the batch effect, the batch allocation and the order of 120 CSF samples and QCs were block-randomized, keeping diagnosis, sex, and age balanced using an in-house R-script. The MP was created by mixing equal volumes from all 120 CSF samples. The CSF used for QC came from a control CSF, separate from the 20 other control CSFs. The MP was divided into each batch after completing the TMT labeling. The QC was divided into 10 batches before reduction and alkylation. Two hundred twenty microliters of each CSF sample were used in this study. All CSF samples, including QC and MP, were prepared by adding 1 volume of 10 M urea in 100 mM triethylammonium bicarbonate (TEAB; Sigma). To perform the reduction and alkylation, 10 mM tris (2-carboxyethyl) phosphine hydrochloride (TCEP; Thermo Scientific) and 40 mM chloroacetamide (CAA; Sigma) were added in the CSF samples and then incubated at room temperature (RT) for 1 h. Protein digestion was carried out using LysC (Lysyl endopeptidase mass spectrometry grade; Fujifilm Wako Pure Chemical Industries Co., Ltd., Osaka, Japan) at the ratio of 1:50 for 3 h at 37 °C and then using trypsin (sequencing grade modified trypsin; Promega, Fitchburg, WI, USA) at the ratio of 1:50 at 37 °C overnight (for 15 h to 18 h) after diluting the concentration of urea from 5 M to 2 M by adding 50 mM TEAB. Peptides were purified using C_18_ Stage-Tips (3 M Empore™;3 M, St. Paul, MN, USA) after acidifying them with trifluoroacetic acid (TFA; Thermo Scientific). The eluted solution containing peptides was vacuum-dried with a Savant SPD121P SpeedVac concentrator (Thermo Scientific). The digested peptides were labeled with 11-plex TMT reagents following the manufacturer’s instructions (Thermo Scientific). MP was labeled by TMT channel 131 C, and the rest of the peptide samples were labeled by one of TMT channels 126, 127 N, 127 C, 128 N, 128 C, 129 N, 129 C, 130 N, 130 C, and 131. The labeling reaction was conducted at RT for 1 h. The remaining TMT tags were quenched by adding 100 mM tris buffer (pH 8.0; Thermo Scientific) and incubating for over 5 min at RT. The peptides for each batch were pooled and subjected to basic pH reversed-phase liquid chromatography (bRPLC) fractionation on an Agilent 1260 HPLC system (Agilent Technologies, Santa Clara, CA, USA). Briefly, the peptides were reconstituted in 10 mM TEAB and fractionated using a bRPLC column (Agilent 300 Extend-C_18_ column, 5 μm, 4.6 mm × 250 mm, Agilent Technologies) under an increasing gradient of the mobile phases consisting of 10 mM TEAB in water and 90% acetonitrile (ACN). A total of 96 fractions were collected by eluting over 97 min (the total run time: 150 min and the collection time: between 50 and 147 min) at a flow rate of 0.3 mL/min and were subsequently concatenated into 24 fractions. The eluted peptides were vacuum-dried.

### LC-MS/MS analysis

The LC-MS/MS analysis was conducted as described in previous publications with minor modifications [16, 17]. The peptide samples were analyzed on an Orbitrap Fusion Lumos Tribrid mass spectrometer interfaced with an Ultimate 3000 RSLCnano nanoflow liquid chromatography system (Thermo Scientific). The fractionated peptides were reconstituted in 0.5% formic acid (FA) and loaded onto a trap column (Acclaim™ PepMap™ 100, LC C_18_, 5 μm, 100 μm × 2 cm, nanoViper, Thermo Scientific) at a flow rate of 8 µL/min. Peptides were separated on an analytical column (Easy-Spray™ PepMap™ RSLC C_18_, 2 μm, 75 μm × 50 cm, Thermo Scientific) at a voltage of about 2.4 kV and at a flow rate of 0.3 µL/min with mobile phases of 0.1% FA in water and in 95% ACN using a linear gradient. The total run time was 120 min. The mass spectrometer was operated in data-dependent acquisition (DDA) mode. The MS1 scan range for a survey full scan was acquired from *m/z* 300 to 1800 in the Orbitrap at a resolution of 120,000 at an *m/z* 200. The automatic gain control (AGC) target for MS1 was set as 1 × 10^6^ and the maximum injection time was set to 50 ms. The most intense ions with charge states of 2 to 5 were isolated in a 3-sec cycle, fragmented using higher-energy collisional dissociation (HCD) fragmentation with 35% normalized collision energy, and detected at a mass resolution of 50,000. The precursor isolation window was set to *m/z* 1.6 with *m/z* 0.4 of offset. The AGC target for MS/MS was set to 5 × 10^4^, and the ion filling time was set to 100 ms. The dynamic exclusion was set to 30 s with a 7 ppm of mass tolerance. Internal calibration was carried out using the lock mass option (*m/z* 445.12002) from ambient air.

### Database searches for peptide and protein identification

Database searches were conducted as described in prior publications with minor modifications [16, 17]. The acquired MS/MS spectra were searched against a human UniProt database (released in May 2018, containing protein entries of common contaminants) using SEQUEST search algorithm in the Thermo Proteome Discoverer platform (version 2.2.0.388, Thermo Scientific). The database search parameters used were as follows. The precursor mass tolerance was set to 10 ppm and the fragment mass tolerance to 0.02 Da. The maximum missed cleavages allowed was 2. Carbamidomethyl (+ 57.02146 Da) at cysteine and TMT tags (+ 229.162932 Da) modification at the N-terminus of a peptide and lysine were set as fixed modifications. Oxidation (+ 15.99492 Da) of methionine was set as a variable modification. The peptides and proteins were filtered at 1% of the false discovery rate (FDR). The protein quantification was performed with the following parameters and methods. Both unique and razor peptides were used for peptide quantification, while protein groups were considered for peptide uniqueness. Reporter ion abundance was computed based on signal-to-noise (S/N) ratios, and the missing intensity values were replaced with the minimum value. The quantification value corrections for isobaric tags were disabled. The average reporter S/N threshold was set to 50. Data normalization was disabled. Protein grouping was performed with a strict parsimony principle to generate the final protein groups. All proteins sharing the same set or subset of identified peptides were grouped, while protein groups with no unique peptides were filtered out. The Proteome Discoverer iterated through all spectra and selected a peptide-spectrum match (PSM) with the highest number of unambiguous and unique peptides.

### Bioinformatics analyses

Gene set enrichment analysis (GSEA) was performed by feeding differentially expressed proteins to the Kyoto encyclopedia of genes and genomes (KEGG) pathway analysis embedded in DAVID Knowledgebase [18, 19]. Interactome analysis was carried out by the Search Tool for the Retrieval of Interacting Genes/Proteins (STRING) protein-protein interaction (PPI) database version 11.5 (https://string-db.org/) [20, 21]. We used a full STRING network to analyze functional and physical protein associations. Cell-type enrichment analysis was conducted as described previously [22]. *P* values for the cell-type enrichment were calculated using Fisher exact tests.

### Experimental design and statistical rationale

Experimental design and statistical analyses were performed as described previously with minor modifications [16, 17]. We conducted sample size analysis using the pwr package in R. When we wanted to detect proteins with > 1.35-fold differences between groups, the required minimum sample size was 31 when the significance level was 0.0001, power was 0.8, sigma was 0.338, and delta was 0.433 (= log_2_ 1.35). The sigma value of 0.338 was derived from our in-house TMT proteomics experiments for the quantification of CSF proteins. We determined the significance level of 0.0001 based on our previous studies. When we identified several thousands of proteins, most of the proteins with *P* value < 0.0001 showed a *q*-value < 0.05. Based on this sample size analysis, we decided to use 40 samples per group. The statistical analysis of the mass spectrometry data was performed with the Perseus version 1.6.0.7 software package. The protein abundance data from 13 batches of the TMT experiments were normalized by dividing the abundance values of each protein by that of MP included in each batch. The relative abundance values for each sample were log2-transformed. We removed proteins with one or more missing values across 120 samples. To further remove batch effects, an additional normalization was conducted with the ComBat package in R. The technical variation was monitored by a coefficient of variation (CV) of QCs embedded in each experimental batch. To estimate CV, the log2-transformed values of the proteins for the QC samples were converted back to the original values, and subsequently, the standard deviation (SD) and mean values of the proteins for the QC samples were determined. The CV was calculated by dividing the SD by the mean. To access the biological variation, the signal-to-noise (S/N) ratio was calculated by dividing the SD estimated from the clinical samples by the SD from the QCs.

Bootstrap receiver operating characteristic (ROC) analysis was carried out using the fbroc package in R. Sampling with replacement was repeated 500 times for the bootstrap ROC. The area under the curve (AUC) of a bootstrap ROC was computed for each sampling. Mean and SD values of AUCs from 500 ROCs were then calculated. This bootstrap ROC was repeated once again after labeling permutation. The *q*-values of bootstrap ROC-based analysis data were calculated as follows: [1] The mean AUC values for non-permuted and permuted data were sorted in descending order for proteins with mean AUCs > 0.5 and in ascending order for proteins with mean AUCs < 0.5; [2] The ratios of the protein numbers for the non-permuted data to the protein numbers for the permuted data were calculated as lowering the cutoff threshold, and the ratios were used as *q*-values.

To assess the classification performance of potential biomarkers, MetaboAnalyst software (version 5.0) was employed through both univariate and multivariate ROCcurve analyses. These analyses were conducted as described previously with minor modifications [23]. For the univariate ROC analysis, a bootstrapping approach involving 500 resampling iterations was implemented to yield an AUC mean value accompanied by a 95% confidence interval. For the multivariate ROC analysis, the partial least squares discriminant analysis (PLS-DA) classification technique, coupled with the inherent feature ranking method of PLS-DA, was used. A total of two latent variables were specified for this analysis. To initiate the multivariate analysis utilizing PLS-DA, ROC curves were generated using balanced subsampling by the Monte-Carlo cross-validation (MCCV) method. In each MCCV iteration, two-thirds of the samples were employed to appraise feature significance, while the remaining one-third served to validate the models developed in the initial phase. Subsequently, the most crucial features were used to construct biomarker classification models. This procedure was reiterated 50 times to estimate the performance metrics and confidence intervals for each respective model. The estimation for the predictive performance was also conducted using the balanced MCCV with 50 iterations, as described above [24]. The average importance, the mean variable importance in projection (mean VIP), of the features was estimated from PLS-DA by subsampling [25]. PCA-biplot was generated using the factoextra package in R.

## Results

### Quantitative proteome analysis of CSF samples

**Fig. 1:**
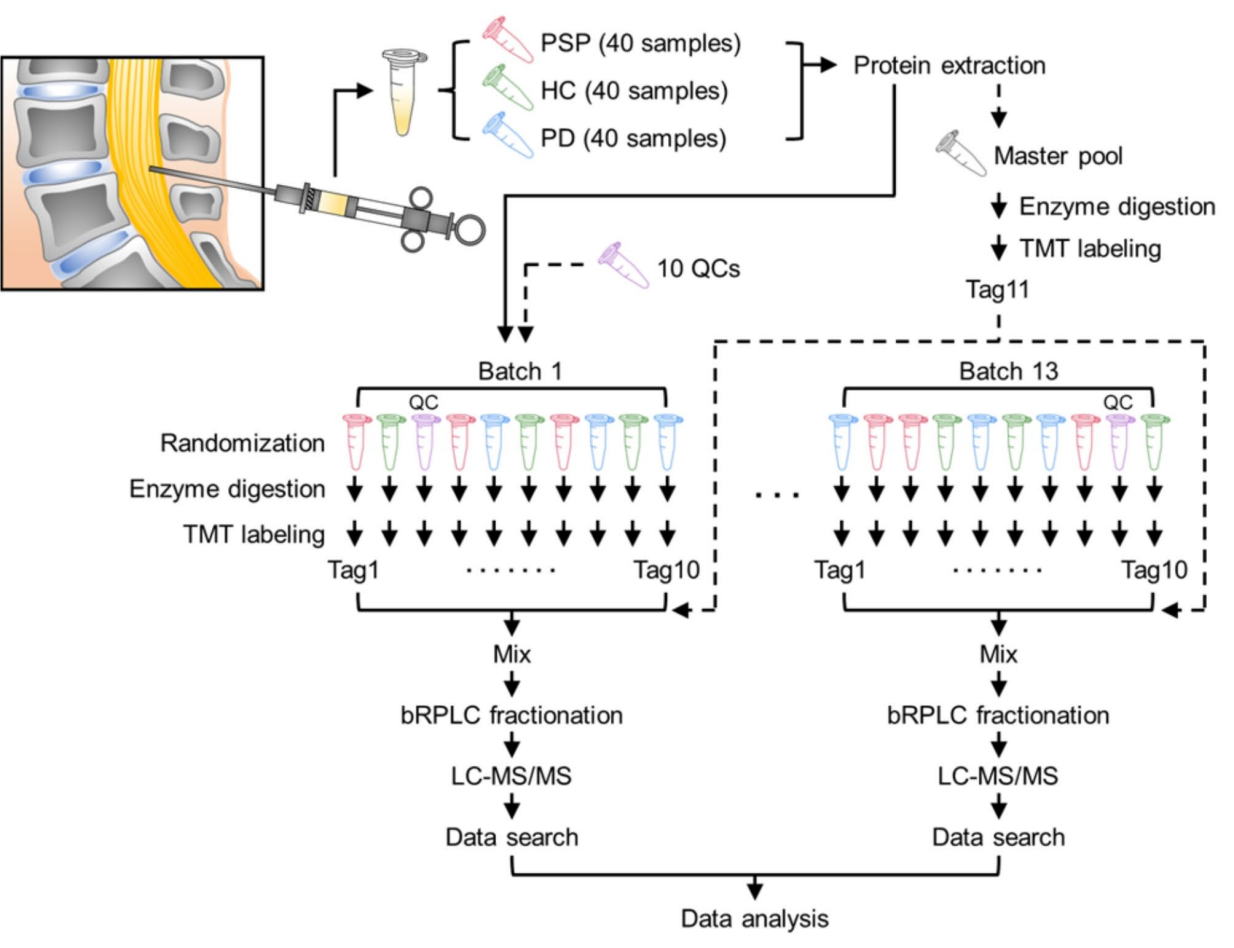
Experimental strategy for the proteomic study of the CSF samples from PSP patients, PD patients, and HC individuals. Thirteen batches of 11-plex TMT experiments were conducted to analyze the proteome of human CSF samples from 40 PSP patients, 40 PD patients, and 40 HC individuals. Master pool (MP) and QC samples were prepared by combining an equal amount of protein from all 120 CSF samples. MP was added to each batch after labeling with Tag 11 in one tube. QC was split into 10 aliquots and processed in 10 of 13 batches separately. TMT tags for individual samples and QC were determined by randomization. The proteins were digested with Lys-C and trypsin, followed by TMT labeling and prefractionation into 24 fractions prior to mass spectrometry analysis. Proteins were identified by conducting a database search of the acquired mass spectra

To identify differentially expressed proteins in PSP, we conducted a quantitative proteome analysis of 120 CSF samples from 40 PSP, 40 PD, and 40 HC individuals. For more accurate quantification of proteins, we exploited the TMT-based quantification method. To analyze 120 CSF samples using 11-plex TMT, we conducted 13 batches of TMT experiments. To normalize the protein abundances between the different batches, we added MP to the last channel of each batch. We also added QC to a random channel in 10 batches each to monitor quantification quality (Fig. 1). We first digested CSF proteins into peptides and then labeled the resulting peptides with TMT tags as described above. For in-depth protein identification, the TMT-labeled peptides were pre-fractionated by bRPLC before mass spectrometry analysis. In total, 23,508,013 MS/MS spectra were acquired, and 2,277,905 MS/MS spectra were assigned to peptides leading to the identification of 283,975 peptides and 3,653 proteins (Supplemental Data S1). The number of proteins that were identified across 13 batches of the TMT experiments was 1,409, which we used for the downstream data analysis (Supplemental Figure S1A). To normalize the data from 13 different batches, the intensity values of each protein were normalized by the MP samples in each batch (Supplemental Figure S1B, left), and then, to remove residual batch effects, another round of normalization was conducted by the ComBat package in R (Supplemental Figure S1B, right). To visually assess the batch effects of 13 batches of the data set before and after the ComBat normalization, the data were plotted on 2D PCA. Batch 2 (orange) showed the biggest batch effect before the Combat normalization, but this batch effect disappeared, and overall data showed a more evenly dispersed pattern. To further assess the quality of the data, the technical variations and S/N ratio of the normalized data were examined (Supplemental Figure S1C). More than 98.7% of proteins manifested technical variations of 20% or less (Supplemental Figure S1C, left). On the other hand, > 99.6% of proteins manifested S/N of 1 or higher, demonstrating the outstanding measurement precision of this TMT-based quantification experiments (Supplemental Figure S1C, right).

### Bootstrap ROC-based statistical analysis for the identification of differential proteins

**Fig. 2:**
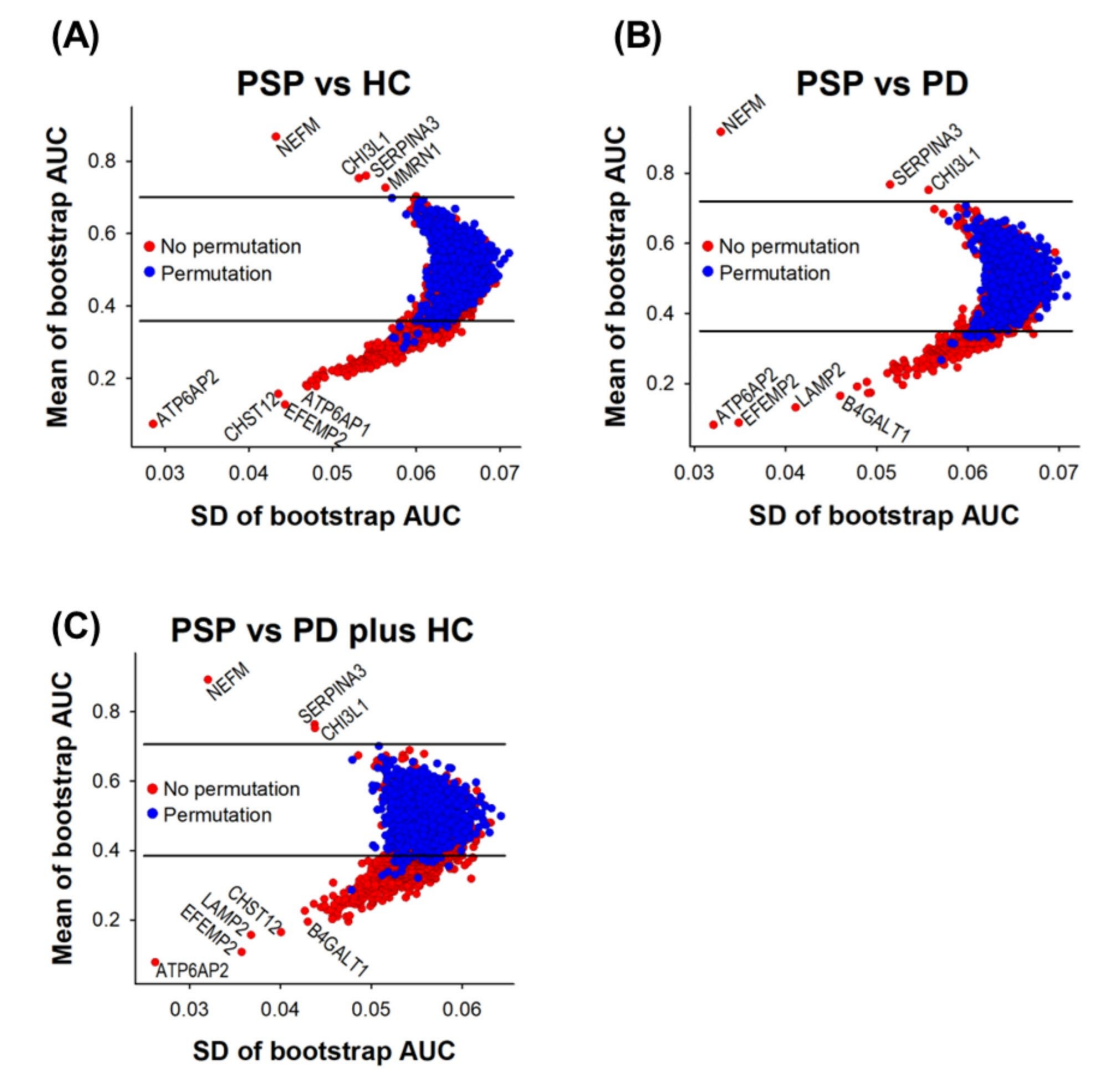
Bootstrap ROC plots of the CSF proteins identified from PSP patients, PD patients, and HC individuals. Bootstrap ROC analyses were conducted to estimate variations of resampling. To calculate *q*-values, bootstrap ROC analyses after permutation of the comparison groups were conducted too. The differentially expressed proteins with a *q*-value < 0.01 are shown at the outside of the upper and lower horizontal lines. The proteins on the upper and lower side of the *q*-value line are up- and down-regulated in PSP compared to HC **(A)**, in PSP compared to PD **(B)** and in PD compared to PD plus HC **(C)**, respectively

We next conducted bootstrap ROC-based statistical analyses to identify proteins differentially expressed in PSP compared to the other groups [16, 17, 26]. For the bootstrap ROC analysis, sampling with replacement was repeated 500 times generating ROC curves for each iteration. This sampling process was repeated once again after a permutation of comparison groups to estimate an FDR. The average AUC and SD of ROC curves were plotted (Fig. 2). When we used a *q*-value of < 0.01 as cutoff lines, the number of differential proteins was 190 between PSP and HC, 152 between PSP and PD, and 247 between PSP and PD plus HC (Supplemental Data S2). When PSP was compared to HC, NEFM was most upregulated followed by CHI3L1, SERPINA3, and MMRN1. On the other hand, ATP6AP2 showed the greatest downregulation, followed by CHST12, EFEMP2, and ATP6AP1 (Fig. 2A). When PSP was compared to PD, a similar pattern was observed. NEFM was the most upregulated, followed by SERPINA3 and CHI3L1, while ATP6AP2 was the most downregulated, followed by EFEMP2, LAMP2, and B4GALT1 (Fig. 2B). When PSP was compared to the group of PD plus HC, a similar pattern was observed. NEFM was the most upregulated, followed by SERPINA3 and CHI3L1, but ATP6AP2 was the most downregulated, followed by EFEMP2, LAMP2, CHST12, and B4GALT1 (Fig. 2C). We summarized the top 50 up- and down-regulated proteins with a *q*-value < 0.01 between PSP and HC (Supplemental Table S1), between PSP and PD (Supplemental Table S2), and between PSP and PD plus HC (Supplemental Table S3). Other than the top differentially expressed proteins, we also observed downregulation of NPTX2 (0.31 of mean of bootstrap AUC and 0.005 of *q*-value in PSP vs. PD plus HC), which is a synaptic protein that plays a crucial role in regulating cortical network dynamics, synaptic adaptability, memory, and is associated with cognitive decline and AD progression (Supplemental Data S2) [27–29]. These results suggest that we successfully identified differentially expressed proteins in PSP.

### Comparison of differentially expressed proteins in CSF with those from Globus Pallidus

The main goal of this study was to discover potential PSP biomarkers. Therefore, we needed to narrow down the list of the differentially expressed proteins in CSF. If the differentially expressed proteins in CSF reflect the changes in the brain, this change should be observed in the brain. Thus, we compared the list of differentially expressed proteins in CSF with the differentially expressed proteins in globus pallidus (GP) of PSP patients, which we reported previously [17]. When PSP was compared to HC, 4 differentially expressed proteins overlapped between CSF and GP (Supplemental Figure S2A). When PSP was compared to PD, only 2 proteins overlapped (Supplemental Figure S2B). When PSP was compared to PD plus HC, 8 proteins were overlapping (Supplemental Figure S2C). CNTNAP2 and EPDR1 were common differentiating proteins for PSP vs. HC and PSP vs. PD plus HC. HAPLN4 was the common differentiating protein for PSP vs. PD and PSP vs. PD plus HC. GGH was the common differentiating protein in all three comparisons (Supplemental Table S4). The limited protein overlap between CSF and GP may be due to the fact that the CSF proteome reflects changes occurring throughout the entire brain during disease progression, whereas the GP proteome represents a specific region of the basal ganglia at the terminal stage of the disease.

### Characterization of differentially expressed proteins in CSF from PSP patients

**Fig. 3:**
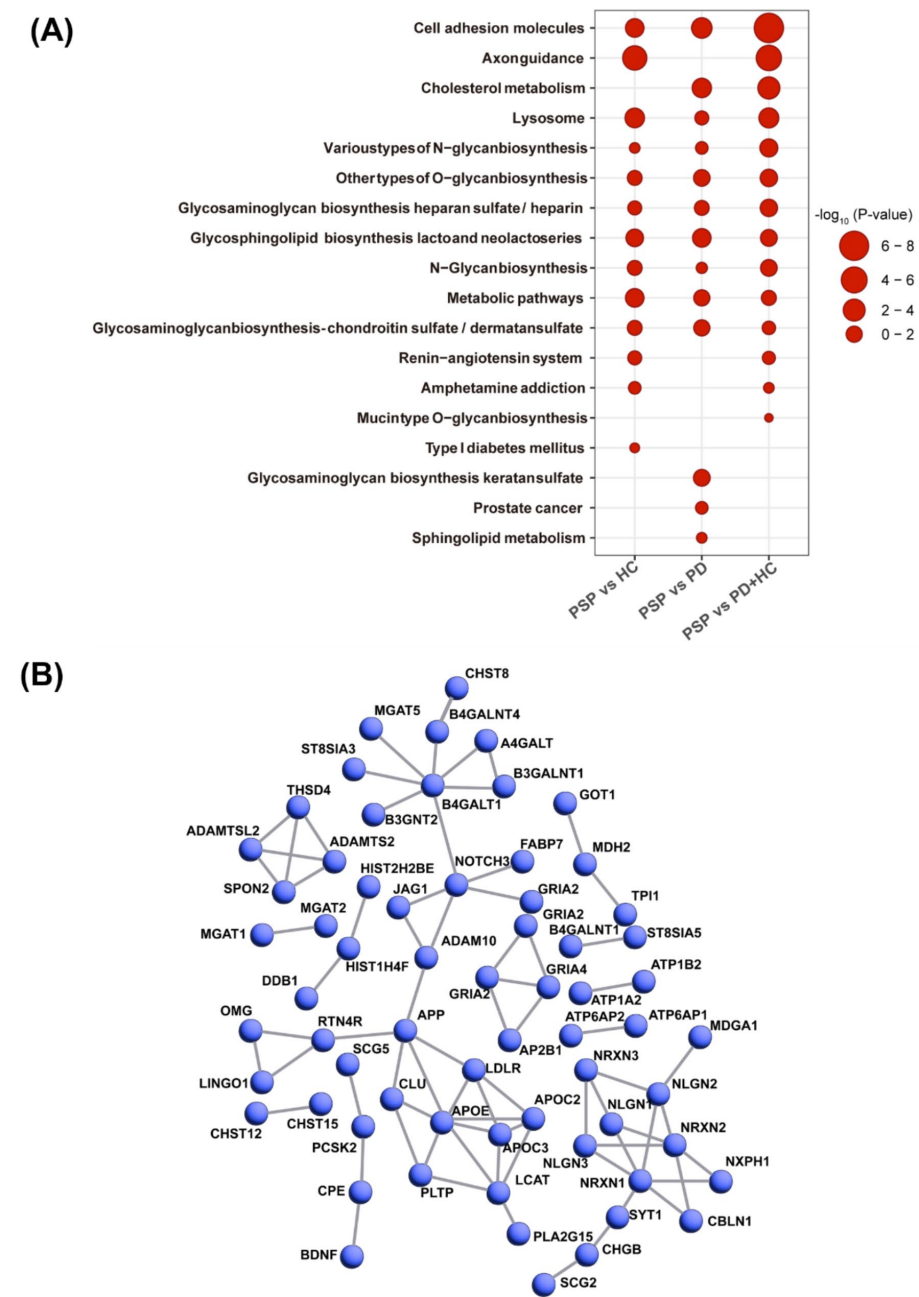
Interactome analysis of differentially expressed proteins. Bubble plot illustrating the –log10 (*P* values) derived from KEGG pathway analysis conducted on the pool of differentially expressed proteins. The vertical axis delineates the pathway names, while the horizontal axis represents the comparative analysis **(A)**. STRING PPI analysis was conducted to estimate the connectivity of the differentially expressed proteins with a *q*-value < 0.01 in PSP compared to the group of HC plus PD. All active interaction sources, including text mining, experiments, databases, co-expression, neighborhood, gene fusion, and co-occurrence, were used with a 0.9 of the highest confidence threshold as a minimum required interaction score. Network edges were set to confidence, which indicates data strength based on thickness. The network contains 241 nodes with 76 edges. (average node degree: 0.63, average local clustering coefficient: 0.178, and PPI enrichment *P-*value < 1 × 10^− 16^). We selected to hide disconnected nodes in the network **(B)**

To better understand the differentially expressed proteins in PSP CSF, we evaluated implicated pathways by GSEA. When PSP was compared to HC, the axonal guidance pathway was the most enriched, followed by lysosome pathway, metabolic pathway, cell adhesion molecules pathway, and glycosphingolipid biosynthesis pathway. When PSP was compared to PD, the cell adhesion molecules pathway was the most enriched, followed by cholesterol metabolism pathway, glycosphingolipid biosynthesis pathway, and glycosaminoglycan biosynthesis pathway. When PSP was compared to the group of PD plus HC, cell adhesion molecules pathway was the most enriched, followed by axonal guidance pathway, cholesterol metabolism pathway, lysosome pathway, and various types of N-glycan biosynthesis pathway (Table 2; Fig. 3A and Supplemental Data S3). As expected, proteins known to be implicated in neurodegeneration represent key components in the enriched pathways. Surprisingly, lipid-related proteins were also frequently observed, suggesting their potential connection to the pathogenesis process of PSP.

**Table 2:** KEGG pathway analysis for the differentially expressed proteins

	Term	Count	%	*P* Value	Benjamini
**PSP vs.** **HC**	Axon guidance	12	6.6	8.E-06	1.30E-03
	Lysosome	8	4.4	9.E-04	6.80E-02
	Metabolic pathways	31	16.9	2.E-03	9.30E-02
	Cell adhesion molecules	8	4.4	2.E-03	9.30E-02
	Glycosphingolipid biosynthesis - lacto and neolacto series	4	2.2	4.E-03	1.20E-01
	Other types of O-glycan biosynthesis	4	2.2	2.E-02	4.40E-01
	N-Glycan biosynthesis	4	2.2	2.E-02	4.40E-01
	Glycosaminoglycan biosynthesis - chondroitin sulfate / dermatan sulfate	3	1.6	2.E-02	4.40E-01
	Renin-angiotensin system	3	1.6	3.E-02	5.00E-01
	Glycosaminoglycan biosynthesis - heparan sulfate / heparin	3	1.6	3.E-02	5.00E-01
	Amphetamine addiction	4	2.2	5.E-02	6.70E-01
	Various types of N-glycan biosynthesis	3	1.6	8.E-02	1.00E + 00
	Type I diabetes mellitus	3	1.6	9.E-02	1.00E + 00
**PSP vs.** **PD**	Cell adhesion molecules	8	5.4	4.E-04	5.60E-02
	Cholesterol metabolism	5	3.4	1.E-03	6.60E-02
	Glycosphingolipid biosynthesis - lacto and neolacto series	4	2.7	2.E-03	7.20E-02
	Glycosaminoglycan biosynthesis - keratan sulfate	3	2	7.E-03	2.10E-01
	Other types of O-glycan biosynthesis	4	2.7	8.E-03	2.10E-01
	Metabolic pathways	23	15.5	1.E-02	2.30E-01
	Glycosaminoglycan biosynthesis - chondroitin sulfate / dermatan sulfate	3	2	1.E-02	2.50E-01
	Glycosaminoglycan biosynthesis - heparan sulfate / heparin	3	2	2.E-02	3.10E-01
	Lysosome	5	3.4	3.E-02	4.20E-01
	Various types of N-glycan biosynthesis	3	2	5.E-02	6.00E-01
	Prostate cancer	4	2.7	5.E-02	6.40E-01
	N-Glycan biosynthesis	3	2	7.E-02	7.80E-01
	Sphingolipid metabolism	3	2	8.E-02	8.00E-01
**PSP vs.** **PD + HC**	Cell adhesion molecules	16	6.6	6.70E-09	1.20E-06
	Axon guidance	14	5.8	2.10E-06	1.90E-04
	Cholesterol metabolism	7	2.9	8.80E-05	5.30E-03
	Lysosome	9	3.7	6.50E-04	2.90E-02
	Various types of N-glycan biosynthesis	5	2.1	3.10E-03	1.10E-01
	Other types of O-glycan biosynthesis	5	2.1	4.70E-03	1.30E-01
	Glycosaminoglycan biosynthesis - heparan sulfate / heparin	4	1.7	4.80E-03	1.30E-01
	Glycosphingolipid biosynthesis - lacto and neolacto series	4	1.7	6.80E-03	1.50E-01
	N-Glycan biosynthesis	5	2.1	7.20E-03	1.50E-01
	Metabolic pathways	33	13.7	1.90E-02	3.50E-01
	Glycosaminoglycan biosynthesis - chondroitin sulfate / dermatan sulfate	3	1.7	3.70E-02	6.10E-01
	Renin-angiotensin system	3	1.2	4.30E-02	6.60E-01
	Amphetamine addiction	4	1.7	7.90E-02	1.00E + 00
	Mucin type O-glycan biosynthesis	3	1.2	9.60E-02	1.00E + 00

A protein-protein interaction analysis was conducted. APOE and B4GALT1 were clustered with 7 other proteins, NRXN1 was clustered with 6 other proteins, and APP, LCAT, NOTCH3, and NRXN2 were clustered with 5 other proteins (Fig. 3B). This interaction analysis suggested that APOE, B4GALT1, NRXN1, NRXN2, APP, LCAT, and NOTCH3 were key components. Among them, APOE, B4GALT1, NRXN1, NRXN2, and LCAT are involved in the cell adhesion molecules pathway, cholesterol metabolism pathway, and glycan biosynthesis pathway in PSP versus PD plus HC of GSEA, further suggesting a potential connection of cell adhesion molecule and cholesterol metabolism pathways to PSP.

Cell-type enrichment analysis was performed to characterize the differentially expressed proteins in PSP compared to the group of PD plus HC. When we analyzed the top 50 up- and down-regulated proteins with a *q*-value < 0.01, astrocytic and neuronal proteins were the most enriched. When we analyzed all the differentially expressed proteins, neuronal proteins were the most enriched, followed by oligodendrocytic and astrocytic proteins (Table 3). These data suggest that neuron-derived proteins were the main component of the proteins that changed in PSP CSF, reflecting the loss of neurons in the PSP brains. On the other hand, astrocytic proteins were the main component among the proteins that changed in greater magnitude in PSP CSF.

**Table 3:** Cell-type-specific enrichment of proteins differential between PSP and PD plus HC

	Cell type	*P* value	List of differential proteins that overlap with the proteins enriched in a specific cell type
**Top 50 up- and down-regulated proteins with ** ***q*** **-value < 0.01**	Astrocyte	0.039	LCAT, TIMP3, CRYM, BTD, SLITRK2, MFGE8, BDNF, PDYN
	Microglia	0.093	LAMP2, CHST12, B4GALT1, ADAM15, GGH, KCTD12, GBA
	Neurons	0.015	NDRG4, MINPP1, ST8SIA5, CDH7, CLSTN3, CHGB, FAT2
	Oligodendrocyte	0.353	PCSK1N, LINGO3, SLITRK4, ADAMTS2, *SERPINA3*
	Endothelia	0.556	B3GNT2, A4GALT, SEMA3G, PREP
**All the differential proteins with ** ***q*** **-value < 0.01**	Astrocyte	0.026	PRDX6, BTD, ALDH1A1 FGFR1, BDNF, MFGE8, PDYN, TRIL, TIMP3, SLITRK2 CRYM, LCAT, ATP1A2, ATP1B2, SCG3, CPE, LINGO1, NOTCH3, NRXN1, APOE, LRIG1, CLU, FABP7, VNN1, NRCAM, EPHB3, EDNRB
	Microglia	0.026	GBA, ASPH, OLFML3, KCTD12, APOC2, ADAM15, GGH, B4GALT1, CHST12, LAMP2, HPRT1, HS6ST1, DPP7, AP1B1, ADGRB1, MGAT2, EPDR1, CORO1A, B4GALNT1 CDH23, LFNG, FAM3A, PLA2G15, MGAT1, PLOD1, CTSA, LIPA
	Neurons	4.68E-11	TRHDE, XXYLT1, NRSN2, ST8SIA5, CLSTN3, CBLN1, ST8SIA3, MINPP1, MGAT5, CDH7, CHGB, NDRG4, FAT2, NPTX1, SCN4B, NRN1, CNTNAP2, EPHA6, PCDHAC2, TMEM59L, NXPH3, SEMA6C, ADGRL1, CBLN4, SCG2, SERPINI1, VGF, L1CAM, RTN4R, RGMB, SCN2B, CA10, CNTNAP5, CAMK2B, MMP24, ROBO2, IDS, CNTNAP4, CACNA2D2, SYT1, CLSTN1, FSTL4, PCSK1, DNER, VWC2, PENK, B4GALNT4, MDGA1, PCDH17, PTPRN2
	Oligodendrocyte	0.009	QPCT, SLITRK4, ADAMTS2, GALNT13, LINGO3, EXTL2, *SERPINA3*, PCSK1N, PCDH7, NFASC, SEMA6A, NPTX2, TMEM132C, SEMA4D, GPR158, NLGN3, ADAM11, ADGRL3, FBLN7, TMEM132D, MFAP4, CA10, LDLR, XYLT1, CHST8, NXPH1, SERPINE2, BRINP1, LRFN2
	Endothelia	0.280	SPOCK2, PREP, SLC39A10 MMRN2, SEMA3G, A4GALT, B3GNT2, HPRT1, GINM1, TFRC, JAG1, ITM2A, ADAMTSL2, PTPRM, SEMA7A, SPON2, FBLN5, GALNT18, PLTP, ART3, ADAM10

Italicized proteins indicate those that showed increased expression in PSP compared to PD plus HC

### Evaluation of the candidate biomarker proteins for classification performance

**Fig. 4:**
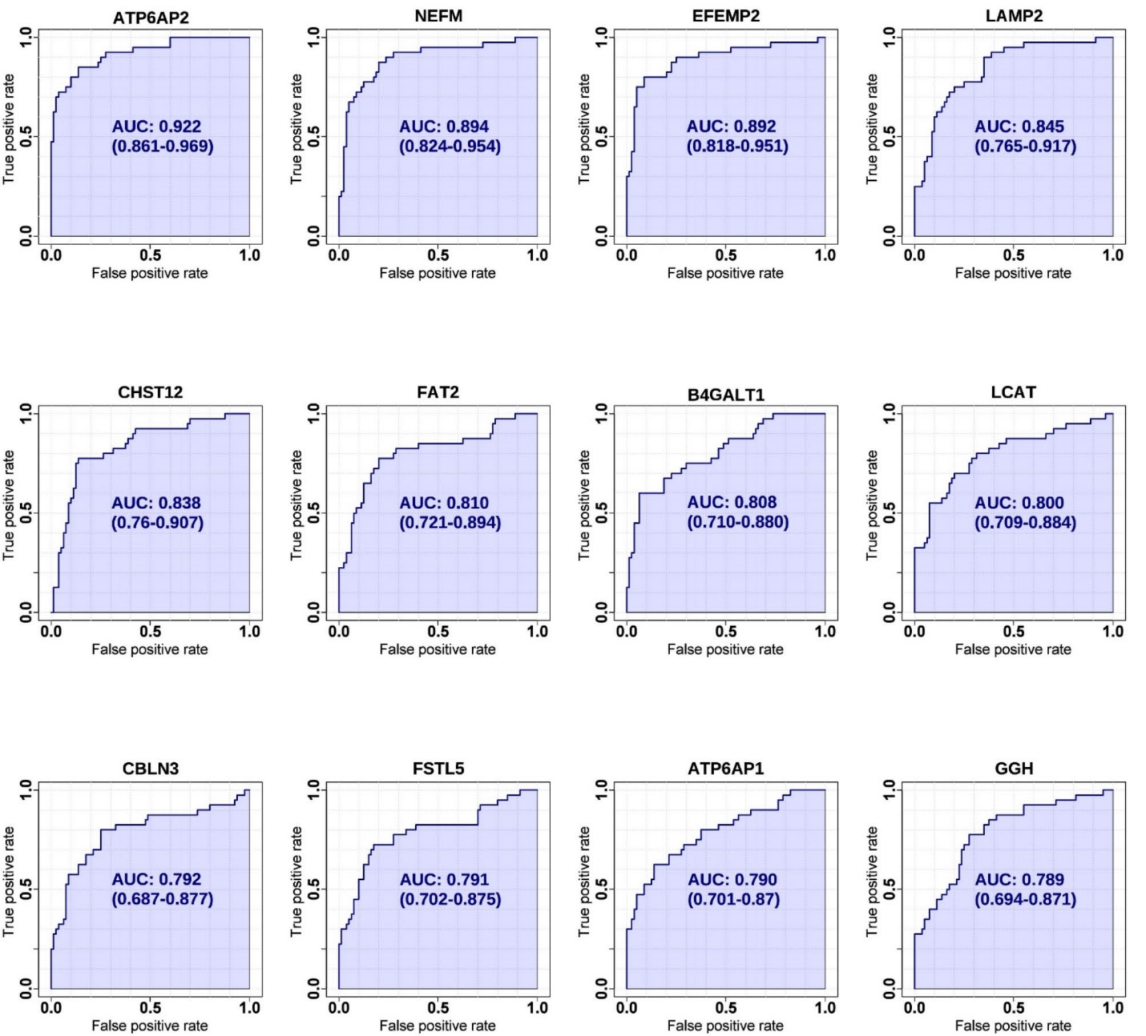
ROC analysis of 12 representative proteins with the highest AUCs between PSP vs. PD plus HC. The discriminating capabilities of candidate PSP biomarkers were estimated by comparing PSP to PD plus HC using ROC analysis. ROC curves were generated by bootstrapping. The values in the parenthesis show the lower and upper AUC values of 95% confidence interval. The values in the parenthesis show the lower and upper AUC values of a 95% confidence interval. The X-axis denotes a false positive rate (1-specificity), and the Y-axis denotes a true positive rate (sensitivity)

**Fig. 5:**
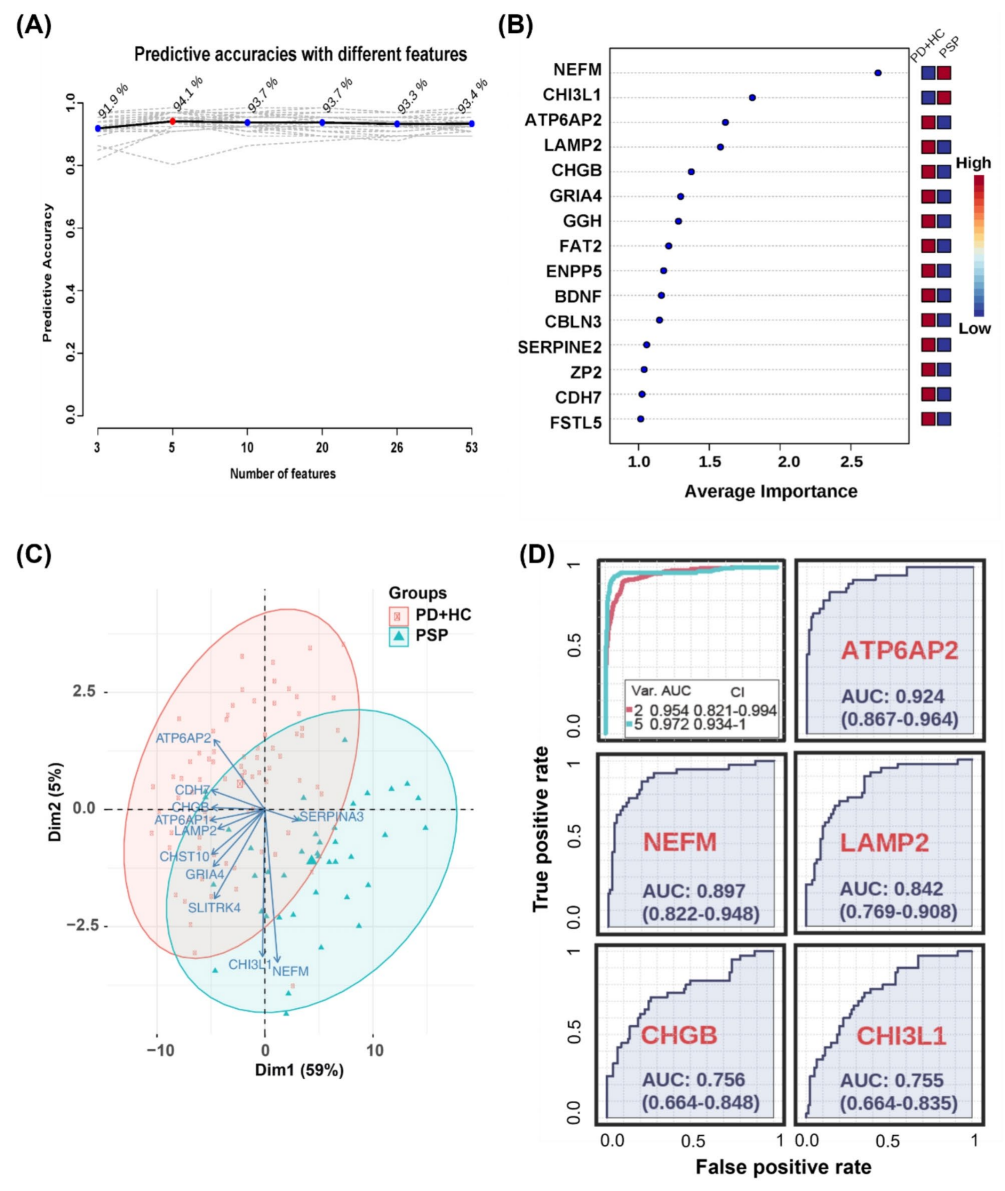
Multivariate ROC analysis and predictive accuracy. The differentially expressed proteins of PSP-specific biomarker candidates were compared with HC plus PD. **(A)** The accuracy for predicting PSP as the number of features increased is shown. **(B)** The top 15 significant features affecting the discrimination of PSP from PD plus HC are shown with their average importance values, which are equivalent to the mean of Variable Importance in Projection (VIP) scores. **(C)** PCA-biplot analysis for the top 53 differential proteins between PSP vs. PD plus HC was conducted. The representative upregulated and downregulated proteins among 53 proteins are shown on the PCA-biplot. **(D)** Multivariate ROC analyses were conducted using 2 and 5 features. Var. indicates the number of features used. CI indicates confidence interval. Individual ROCs for 5 proteins used for the multivariate ROC are shown too.

As the main goal of this study was to identify proteins that can be used to differentiate PSP from HC and PD, we evaluated the classification performance of differentially expressed proteins using ROC analysis. ATP6AP2 showed the highest AUC value (0.922), followed by NEFM (AUC 0.894), EFEMP2 (AUC 0.892), LAMP2 (AUC 0.845), CHST12 (AUC 0.838), FAT2 (AUC 0.810), B4GALT1 (AUC 0.808), LCAT (AUC 0.800), CBLN3 (AUC 0.792), FSTL5 (AUC 0.791), ATP6AP1 (AUC 0.790), and GGH (AUC 0.789) (Fig. 4). The remainder of the top 50 up- and down-regulated proteins with a *q*-value < 0.01 showed AUC > 0.696 (Supplemental Figure S3). To further improve the classification performance of differentially expressed proteins, we conducted multivariate analyses by varying the number of features up to 53. The 53 features for the multivariate analysis were from the top 50 up- and down-regulated proteins with a *q*-value < 0.01, when comparing PSP versus PD plus HC (Supplemental Table S3). The predictive accuracy reached the maximum value, 94.1%, when 5 features were used. After then, the predictive accuracy slightly decreased when more features were used, suggesting that it was overfitted when more features were used (Fig. 5A). NEFM was the most contributing marker followed by CHI3L1, ATP6AP2, LAMP2, CHGB, GRIA4, GGH, FAT2, ENPP5, BDNF, CBLN3, SERPINE2, ZP2, CDH7, and FSTL5 (Fig. 5B). To estimate how these marker proteins contributed to discriminating the PSP group from the other two groups, we conducted a PCA-biplot analysis. The PCA-biplot showed why it was overfitted when more than 5 features were used. The marker proteins formed a few clusters: ATP6AP2 and CDH7 that contributed to the negative direction for dimension 1 and the positive direction for dimension 2; CHGB and ATP6AP1 that contributed to the negative direction only for dimension 1; LAMP2, CHST10, GRIA4, and SLITRK4 that contributed to the negative direction for dimensions 1 and 2; CHI3L1 and NEFM that contributed to the negative direction only for dimension 2; and SERPINA3 that contributed to the positive direction only for dimension 1. Among them, NEFM, ATP6AP2, and CHI3L1 contributed the most to discriminating the PSP group from the other two groups. CDH7, CHGB, and SERPINA3 were complementary to the three most contributing proteins while the discriminating powers of the individual proteins were weaker (Fig. 5C). So, we conducted multivariate ROC analysis using the top 5 important marker proteins. While the AUCs for the individual proteins were 0.924 or lower, the AUC of the multivariate analysis using the 5 marker proteins was increased to 0.972 (Fig. 5D). NEFM showed the highest average importance, followed by LAMP2, ATP6AP2, CHGB, and CHI3L1 when both 5 and 2 features were used in the classification model (Supplemental Figure S4) These data suggest that the combination of these potential biomarker proteins can be used to marginally improve the classification performance, and NEFM, CHI3L1, and ATP6AP2 are key proteins in differentiating PSP from two other groups, although a further validation experiment is required.

## Discussion

In this study, mass spectrometry-based proteomic analysis of 120 human CSF samples from 40 PSP, 40 PD, and 40 HC individuals was conducted using the TMT-based multiplexing approach, identifying 3,653 proteins. Although we analyzed 120 CSF samples using 13 batches of 11-plex TMT experiments, the precision of the experiment was very high, with < 10% of CV for most of the proteins. This suggests that the two-step normalization using MP and, subsequently, the ComBat package was effective for analyzing the large number of CSF samples using the TMT-based quantification approach.

Since we wanted to explore whether the differentially expressed proteins in both CSF and GP of PSP patients may have greater relevance as PSP biomarkers, we built on our prior work and compared the proteins differentially expressed in both CSF and GP. While ATP1B2, CNTNAP2, EPDR1, FBLN2, GGH, GOT1, HAPLN4, PREP, and SERPINE2 were the main differentially expressed proteins common in CSF and GP of PSP patients, they were not identified as the key proteins in GSEA, interactome analysis, and ROC analyses. This discrepancy suggests that GP-derived proteins may not reflect the same pathogenic process giving rise to differentially expressed proteins in PSP CSF. Rather, because multiple other brain regions (such as the subthalamic nucleus, substantia nigra, putamen, and perirolandic cortex) are prominently affected by PSP pathology, evaluating protein expression in these additional regions may yield results in greater concordance with the CSF findings [30, 31]. Furthermore, because autopsy samples are mostly derived from patients in advanced stages of PSP, the differentially expressed proteins in early or mid-stage PSP could well be different from the ones expressed in the advanced stages—reflecting a dynamic pathophysiological process. Further investigations utilizing CSF samples derived from multiple disease stages and autopsy samples derived from multiple implicated brain regions are necessary to further characterize the proteomic signature of PSP.

GSEA and interactome analysis demonstrated that cell adhesion molecules pathway, cholesterol metabolism pathway, and glycan biosynthesis pathway were the critical ones for the differentially expressed proteins in PSP CSFs. In these pathways, APOE, B4GALT1, NRXN1, NRXN2, and LCAT were key proteins. Cell adhesion molecules are already known to be involved in neurodegenerative diseases [32, 33], especially by altering synaptic plasticity, neuroinflammatory events, and effecting vascular changes. Cholesterol is an indispensable component of the cell membrane, and aberrations of cholesterol metabolism are involved in various neurodegenerative conditions, including Alzheimer’s disease (AD) and PD [34]. Glycan is a key molecule involved in the modification of lipids, proteins, and other glycans [35]. Glycosylated lipids are involved in cell adhesion and glycosylated proteins are major components of cell membrane proteins [36]. Our cell-type enrichment analysis results indicated that the main fraction of the differentially expressed proteins was derived from neuronal cells, suggesting that the pathway changes observed in CSF were predominantly from neuronal cells; further investigation is required to validate this.

The primary aim of this study was to identify biomarkers for PSP. To this end, we assessed the discriminatory potential of several candidate biomarker proteins for PSP. ATP6AP2 had the highest AUC, followed by NEFM, EFEMP2, LAMP2, CHST12, FAT2, B4GALT1, LCAT, CBLN3, FSTL5, ATP6AP1, and GGH, when compared to PD and HC. Of the top 12 proteins, B4GALT1 plays a role in glycan biosynthesis, while LCAT is involved in cholesterol metabolism. Both these proteins emerged as significant in our interactome analysis as well, suggesting that B4GALT1 and LCAT might be promising novel biomarkers for PSP.

B4GALT1 is a galactosyltransferase enzyme, which is responsible for the synthesis of oligosaccharides in glycoproteins and glycolipids. B4GALT1 is known to be linked to microglial activation and neuroinflammation [37]. In AD brains, elevated B4GALT1 expression correlated with heightened galactosylation of N-glycans [38]. Furthermore, a previous study indicated a notable increase in B4GALT1 gene expression within the substantia nigra of PD patients compared to controls [37]. However, our result showed downregulation of B4GALT1 in the CSF from PSP patients when compared to PD and HC. Further investigation is required to assess whether and how B4GALT1 is involved in the pathogenesis of PSP.

LCAT is a lipoprotein-associated enzyme that plays a key role in transferring excessive cholesterol in peripheral tissues to the liver for excretion [39, 40]. The dysregulation of LCAT leads to the disturbance of lipid metabolism and it is potentially implicated in the pathogenesis of PD [41]. Recent plasma metabolomics analyses underscore this by revealing a decrease in lipid and lipid-associated molecules in PD compared to the control group [42]. Our findings showed that LCAT was downregulated in PSP patients compared to PD and HC, suggesting the link between lipid metabolism disturbance by LCAT dysregulation and PSP pathogenesis.

ATP6AP2 is ATPase H + transporting lysosomal accessory protein, which is a vital component of the vacuolar ATPase and plays a crucial role in lysosomal functions and autophagy. Deficiency in ATP6AP2 disrupts V-ATPase function, affecting neural stem cell renewal and causing widespread neural degeneration, emphasizing ATP6AP2’s central role in the developing human nervous system [43]. The dysregulation of ATP6AP2 was also reported to be implicated in Parkinsonism [44]. Our findings indicate a decreased expression of ATP6AP2 in PSP patients relative to both HC and PD, suggesting that ATP6AP2 dysregulation plays a role in PSP. Notably, the mode of ATP6AP2 dysregulation in PSP appears distinct from that in PD, given the differential levels observed between the two patient groups. Further study is required to investigate this distinction.

Neurofilament proteins, including NEFM, are considered promising candidate state biomarkers for neuronal damage and the process of neurodegeneration [45]. However, they are relatively non-specific when attempting to differentiate among neurological diseases diagnostically. Elevated levels within CSF have been demonstrated for patients with stroke and a wide spectrum of neurodegenerative and neuroinflammatory conditions [46, 47]. While there is a lack of research on the relationship between neurofilament proteins and PSP, our findings suggest that PSP patients sustain significant ongoing neuronal damage and thus release greater amounts of NEFM into CSF compared to PD and HC individuals.

EFEMP2, also known as fibulin-4, is a member of the fibulin glycoprotein family found predominantly in elastic fiber-rich tissues and is vital for elastic fiber formation, connective tissue development, and extracellular matrix stability [48]. EFEMP2 has been reported to have implications in the advancement of different cancer types [49]. Little is known about the relationship between EFEMP2 and neurodegeneration. We found downregulation of EFEMP2 in PSP patients compared to HC and PD, and further investigation of this relationship is required.

LAMP2 is a lysosomal-associated membrane protein and constitutes a significant portion of the lysosomal membrane [50]. Lysosomes serve as the main catabolic units responsible for breaking down intracellular proteins via the process of autophagy [51]. The existence of α-synuclein aggregates in PD is potentially mediated by compromised degradation capabilities of lysosomes [52]. A prior investigation using Western blot quantification reported that PD CSFs showed reduced levels of LAMP2 compared to HC, while PSP CSF did not show differences [53]. Our result showed a downregulation of LAMP2 in PSP patients compared to HC and PD. This discrepancy could be caused by quantification method differences or case specificity, and further investigation is required to clarify this.

CHST12 is a carbohydrate sulfotransferase involved in the biosynthesis of proteoglycans that facilitate cell interactions. Its overexpression serves as an unfavorable prognostic factor in ovarian cancer [54]. Little is known about the involvement of CHST12 in neurodegeneration. We found that the CHST12 level was decreased in PSP compared to PD and HC.

FAT2 is a cadherin superfamily protein and is known to be expressed in granule cells in the cerebellum [55]. The cadherin family proteins have consistently demonstrated their influence in governing the contact between axons and dendrites [56]. Our results showed a downregulation of FAT2 in PSP patients compared to PD and HC. Further investigation is required to understand how FAT2 is involved in PSP.

CBLN3 is a member of the precerebellin protein family [57] and is expressed in cerebellum and dorsal cochlear nucleus [57]. The link between CBLN3 and neurodegeneration is not clear, although our finding shows that CBLN3 was downregulated in PSP compared to PD and HC and cerebellar pathology (particularly in the dentate) is well-described in PSP [58, 59]. 

FSTL5 is a secretory glycoprotein [60] and is known to be a prognostic biomarker for medulloblastoma [61]. Our data showed that FSTL5 was significantly downregulated in PSP compared PD and HC.

ATP6AP1 is an accessory protein of V-type ATPase proton pump [62]. Its role is to direct the V-ATPase to specific subcellular compartments, such as neuroendocrine-regulated secretory vesicles, and to regulate various aspects of their function, including intragranular pH and the Ca2+-dependent exocytotic membrane fusion [48]. In our results, ATP6AP1 showed significant downregulation in the PSP compared to PD and HC. Considering that both ATP6AP1 and ATP6AP2 are downregulated in PSP, the subcellular mislocalization of V-type ATPase proton pump by the dysfunction of its accessory proteins is potentially involved in PSP pathogenesis.

GGH is an enzyme involved in folate metabolism [63]. Fang et al. reported GGH was downregulated in human CSF from Huntington disease patients [64]. Licker et al. also reported that GGH was downregulated in substantia nigra of PD patients [65]. Our finding also showed that GGH was significantly downregulated in PSP compared to PD and HC. These studies suggest that GGH is downregulated in multiple various neurodegenerative diseases.

In this study, NPTX2 was downregulated in PSP compared to PD and HC. The downregulation of NPTX2 is a predictive marker for the progression from normal cognition to mild cognitive impairment [27], and cognitive decline is a typical symptom of PSP [66]. This suggests that dysregulated synaptic adaptability mediated by NPTX2 downregulation could be a potential mechanism of the cognitive decline of PSP patients.

Multivariate analysis showed marginally improved discriminating capability (AUC 0.937) compared to the best single-marker AUC (0.922) of ATP6AP2. This suggests that ATP6AP2 is a promising single-marker candidate for PSP and that integrating multiple PSP biomarkers could be beneficial for the better diagnosis of PSP. Interestingly, CHI3L1, which has a relatively lower AUC (0.755), was selected as the second most important feature in the multivariate analysis. CHI3L1 was the only protein that had a similar loading value to that of NEFM, while most other proteins had similar loading values to that of ATP6AP2. Thus, CHI3L1 had a high average importance because of its high complementarity with other proteins.Important study limitations include the lack of post-mortem confirmation of PSP or PD diagnosis and differences between groups with respect to age, race/ethnicity, and education. Every effort to match samples on demographic characteristics was made, but we acknowledge that these differences may have contributed to differential CSF protein expression in ways that are not currently well understood. It should be noted that lower education levels have previously been associated with higher likelihood of a PSP diagnosis [67], though the pathophysiological mechanism of this association remains unclear. The candidate biomarkers discovered in this study also need to be validated using an independent cohort and also evaluated for their applicability to differentiate across subtypes of PSP.

## Conclusion

To the best of our knowledge, this is the first global-scale proteome analysis to discover CSF PSP biomarkers using a mass spectrometry-based proteomics approach and utilizing samples from well-matched PSP, PD, and HC. The biomarker candidate proteins ATP6AP2, NEFM, and LAMP2 were identified as key differentiators of PSP from the other groups. The identification of these key differentially expressed proteins and their associated pathways provides a crucial foundation for the development and validation of specific, reliable biomarkers for PSP diagnosis.

## Electronic supplementary material

Below is the link to the electronic supplementary material.

Supplementary Material 1

Supplementary Material 2

Supplementary Material 3

Supplementary Material 4

## Data Availability

The mass spectrometry data from this study have been deposited to the ProteomeXchange Consortium (https://www.proteomexchange.org) via PRIDE partner repository with the dataset identifier ‘PXD041417’, project name ‘Biomarkers discovery for progressive supranuclear palsy from the human cerebrospinal fluid using mass spectrometry-based proteomics.’ Reviewers can access the dataset by using ‘reviewer_pxd041417@ebi.ac.uk’ as ID and ‘kdIwetsc’ as a password.

## Abbreviations

ACN: Acetonitrile
AGC: Automatic gain control
AUC: Area under the curve
bRPLC: Basic pH reversed-phase liquid chromatography
CAA: Chloroacetamide
CSF: Cerebrospinal fluid
CV: Coefficient variation
DDA: Data-dependent acquisition
FA: Formic acid
FDR: False-discovery rate
HC: Healthy control
HCD: Higher-energy collisional dissociation
KEGG: Kyoto encyclopedia of genes and genomes
m/z: mass-to-charge ratio
MP: Master pool
PD: Parkinson’s disease
PSM: Peptide-spectrum match
PSP: Progressive supranuclear palsy
QC: Quality control
ROC: Receiver operating characteristic
RT: Room temperature
S/N: Signal-to-noise ratios
SD: Standard deviation
TCEP: Tris (2-Carboxyethyl) phosphine hydrochloride
TEAB: Triethylammonium bicarbonate
TFA: Trifluoroacetic acid
TMT: Tandem mass tag
