# Supplementary material for: Biomarker discovery in progressive supranuclear palsy from human cerebrospinal fluid
Source: Clin Proteomics. 2024 Sep 28;21:56. doi: 10.1186/s12014-024-09507-3 (PMC11437921; doi:10.1186/s12014-024-09507-3)
Supplement: Supplementary file 3 — Supplementary Material 3 [file 12014_2024_9507_MOESM3_ESM.docx]

**Supplemental Data for**

**Biomarker discovery in progressive supranuclear palsy from human cerebrospinal fluid**

Yura Jang^#^, Sungtaek Oh^#^, Anna J. Hall, Zhen Zhang, Thomas F. Tropea, Alice Chen-Plotkin, Liana S. Rosenthal, Ted M. Dawson^*^, Chan Hyun Na^*^, and Alexander Y. Pantelyat^*^

^#^These authors contributed equally to this work.

*Corresponding author e-mail: [tdawson@jhmi.edu](mailto:tdawson@jhmi.edu) (T.M.D), [chanhyun@jhmi.edu](mailto:chanhyun@jhmi.edu) (C.N), and [apantel1@jhmi.edu](mailto:apantel1@jhmi.edu)(A.Y. P)

**Table of Contents**

Supplemental Table S1. Top 50 up- and down-regulated differential proteins with a q-value < 0.01 in PSP compared to HC. ………………………………………………………………………………………... **S-2**

Supplemental Table S2. Top 50 up- and down-regulated differential proteins with a q-value < 0.01 in PSP compared to PD. ……………………………………………………………………………………...… **S-3**

Supplemental Table S3. Top 50 up- and down-regulated differential proteins with a q-value < 0.01 in PSP compared to PD plus HC………………………………………………………………………...………**S-4**

Supplemental Table S4. Overlapping differentially expressed proteins between PSP GP and PSP CSF. .**S-5**

Supplemental Figure S1. The evaluation of proteomic data quality from 13 batches of 11-plex TMT experiments. ………………………………………………………………………………………..…….**S-6**

Supplemental Figure S2. Comparison of differentially expressed proteins in CSF of PSP with the ones in GP of PSP. ………………………………………………………………………………………….…….**S-7**

Supplemental Figure S3. ROC analyses for the candidate PSP biomarker proteins. ...……………**S-8, S-9**

Supplemental Figure S4. Average importance of features of the multivariate analyses using the top 5 features……………………………………………………………………………………………..…**S-10**

**Supplemental Table S1. Top 50 up- and down-regulated differential proteins with a q-value < 0.01 in PSP compared to HC.**

| **No.** | **Protein**  **name** | **P value** | **Mean of**  **bootstrap AUC** | **SD of**  **bootstrap AUC** | ***q*-value** |
| --- | --- | --- | --- | --- | --- |
| **Down-regulated proteins** | | | | | |
| 1 | ATP6AP2 | 4.97E-14 | 0.073750 | 0.0285685 | 0 |
| 2 | CHST12 | 2.66E-08 | 0.156875 | 0.0435088 | 0 |
| 3 | EFEMP2 | 4.46E-10 | 0.126875 | 0.0443257 | 0 |
| 4 | LAMP2 | 1.34E-07 | 0.181250 | 0.0468534 | 0 |
| 5 | ATP6AP1 | 5.35E-08 | 0.176875 | 0.0470242 | 0 |
| 6 | PCMT1 | 4.86E-07 | 0.193750 | 0.0474892 | 0 |
| 7 | NDRG4 | 2.96E-06 | 0.208750 | 0.0480604 | 0 |
| 8 | FSTL5 | 1.92E-07 | 0.178125 | 0.0480624 | 0 |
| 9 | FAT2 | 6.77E-07 | 0.194375 | 0.0481374 | 0 |
| 10 | FBLN2 | 9.20E-06 | 0.221250 | 0.0488661 | 0 |
| 11 | CBLN3 | 2.82E-06 | 0.206250 | 0.0489301 | 0 |
| 12 | PCSK2 | 3.35E-06 | 0.208750 | 0.0489551 | 0 |
| 13 | ZP2 | 1.84E-06 | 0.201875 | 0.0490643 | 0 |
| 14 | B3GNT2 | 4.02E-06 | 0.221250 | 0.0499495 | 0 |
| 15 | CLSTN3 | 2.69E-06 | 0.216250 | 0.0506021 | 0 |
| 16 | SEMA3G | 1.05E-05 | 0.224375 | 0.0506108 | 0 |
| 17 | CDH7 | 6.47E-06 | 0.229375 | 0.0507745 | 0 |
| 18 | ANGPTL2 | 3.70E-06 | 0.218125 | 0.0509816 | 0 |
| 19 | B4GALT1 | 6.02E-06 | 0.224375 | 0.0513025 | 0 |
| 20 | MFGE8 | 9.75E-06 | 0.223750 | 0.0514011 | 0 |
| 21 | PREP | 5.02E-06 | 0.213750 | 0.0515128 | 0 |
| 22 | LINGO3 | 1.15E-05 | 0.229375 | 0.0517718 | 0 |
| 23 | PCSK1N | 1.27E-06 | 0.205625 | 0.0517753 | 0 |
| 24 | BDNF | 5.46E-06 | 0.221250 | 0.0520363 | 0 |
| 25 | GGH | 2.33E-05 | 0.231250 | 0.0521141 | 0 |
| 26 | GOT1 | 4.12E-05 | 0.253750 | 0.0521524 | 0 |
| 27 | ACP2 | 3.69E-05 | 0.236250 | 0.0522678 | 0 |
| 28 | LCAT | 9.67E-06 | 0.229375 | 0.0523143 | 0 |
| 29 | ENPP5 | 1.45E-05 | 0.222500 | 0.0523766 | 0 |
| 30 | OLFML3 | 1.67E-05 | 0.222500 | 0.0524193 | 0 |
| 31 | ADAM15 | 3.02E-05 | 0.238437 | 0.0524369 | 0 |
| 32 | MGAT5 | 5.03E-06 | 0.215625 | 0.0525166 | 0 |
| 33 | TPI1 | 2.45E-05 | 0.243750 | 0.0527149 | 0 |
| 34 | CRYM | 9.85E-06 | 0.241250 | 0.0528349 | 0 |
| 35 | EXTL2 | 3.50E-06 | 0.225625 | 0.0528380 | 0 |
| 36 | XXYLT1 | 3.70E-05 | 0.239375 | 0.0530386 | 0 |
| 37 | QSOX2 | 9.12E-05 | 0.244375 | 0.0533785 | 0 |
| 38 | GALNT13 | 4.72E-05 | 0.243750 | 0.0533830 | 0 |
| 39 | ST8SIA3 | 3.56E-06 | 0.226250 | 0.0534163 | 0 |
| 40 | FAM3A | 3.42E-05 | 0.248750 | 0.0534198 | 0 |
| 41 | MINPP1 | 5.10E-05 | 0.251875 | 0.0534487 | 0 |
| 42 | CHGB | 1.08E-05 | 0.232500 | 0.0536000 | 0 |
| 43 | NUTF2 | 3.65E-05 | 0.250000 | 0.0536198 | 0 |
| 44 | EPHA6 | 3.76E-05 | 0.251250 | 0.0536506 | 0 |
| 45 | CA11 | 8.76E-06 | 0.228125 | 0.0538124 | 0 |
| 46 | SRPX | 8.47E-05 | 0.244375 | 0.0538150 | 0 |
| 47 | TIMP4 | 1.37E-05 | 0.234375 | 0.0539270 | 0 |
| 48 | ARL8B | 1.57E-05 | 0.238125 | 0.0540940 | 0 |
| 49 | SPOCK2 | 1.76E-05 | 0.242500 | 0.0540950 | 0 |
| 50 | SLC39A10 | 7.67E-05 | 0.259375 | 0.0541175 | 0 |
| **Up-regulated proteins** | | | | | |
| 1 | NEFM | 8.17E-10 | 0.867500 | 0.0432413 | 0 |
| 2 | CHI3L1 | 5.45E-05 | 0.753125 | 0.0531420 | 0 |
| 3 | SERPINA3 | 2.40E-05 | 0.760000 | 0.0539946 | 0 |
| 4 | MMRN1 | 1.08E-03 | 0.726875 | 0.0563351 | 0 |
| 5 | SELL | 2.39E-03 | 0.702500 | 0.0599897 | 0 |

**Supplemental Table S2. Top 50 up- and down-regulated differential proteins with a q-value < 0.01 in PSP compared to PD.**

| **No.** | **Protein**  **name** | **P value** | **Mean of**  **bootstrap AUC** | **SD of**  **bootstrap AUC** | ***q*-value** |
| --- | --- | --- | --- | --- | --- |
| **Down-regulated proteins** | | | | | |
| 1 | ATP6AP2 | 1.19E-13 | 0.081875 | 0.032083 | 0 |
| 2 | EFEMP2 | 1.92E-11 | 0.088125 | 0.034850 | 0 |
| 3 | LAMP2 | 2.61E-10 | 0.131875 | 0.041099 | 0 |
| 4 | B4GALT1 | 1.46E-08 | 0.165000 | 0.045994 | 0 |
| 5 | GGH | 9.38E-07 | 0.191250 | 0.047841 | 0 |
| 6 | APOC2 | 3.88E-06 | 0.204375 | 0.048907 | 0 |
| 7 | CHST12 | 4.74E-07 | 0.172500 | 0.049057 | 0 |
| 8 | LCAT | 1.87E-07 | 0.173750 | 0.049328 | 0 |
| 9 | FBLN2 | 8.40E-06 | 0.229375 | 0.051174 | 0 |
| 10 | MINPP1 | 2.12E-05 | 0.240000 | 0.051796 | 0 |
| 11 | CHGB | 7.93E-05 | 0.255625 | 0.051828 | 0 |
| 12 | APOC3 | 5.12E-05 | 0.243125 | 0.052078 | 0 |
| 13 | ADAM15 | 1.95E-05 | 0.234375 | 0.052089 | 0 |
| 14 | PCMT1 | 3.14E-05 | 0.249375 | 0.052186 | 0 |
| 15 | FGFR1 | 3.59E-05 | 0.244375 | 0.052191 | 0 |
| 16 | CBLN3 | 5.35E-06 | 0.211875 | 0.052227 | 0 |
| 17 | THSD4 | 8.84E-06 | 0.227500 | 0.052273 | 0 |
| 18 | GRIA4 | 6.15E-05 | 0.240625 | 0.052510 | 0 |
| 19 | FAT2 | 1.61E-06 | 0.195625 | 0.052850 | 0 |
| 20 | ST8SIA3 | 1.30E-04 | 0.266250 | 0.052959 | 0 |
| 21 | SELENOF | 1.66E-04 | 0.254375 | 0.053081 | 0 |
| 22 | ZP2 | 1.13E-04 | 0.255625 | 0.053125 | 0 |
| 23 | KCTD12 | 2.98E-06 | 0.222500 | 0.053187 | 0 |
| 24 | ATP6AP1 | 1.58E-05 | 0.243125 | 0.053334 | 0 |
| 25 | A4GALT | 8.73E-05 | 0.240000 | 0.053431 | 0 |
| 26 | NDRG4 | 3.48E-05 | 0.243125 | 0.054273 | 0 |
| 27 | CRYM | 1.42E-04 | 0.264375 | 0.054415 | 0 |
| 28 | C1QTNF3-AMACR | 1.37E-05 | 0.223750 | 0.054418 | 0 |
| 29 | CHST10 | 4.91E-05 | 0.255625 | 0.055019 | 0 |
| 30 | ST8SIA5 | 4.80E-05 | 0.241875 | 0.055036 | 0 |
| 31 | NOMO2 | 2.51E-04 | 0.270000 | 0.055097 | 0.006711 |
| 32 | NRXN2 | 2.90E-04 | 0.276250 | 0.055463 | 0.006711 |
| 33 | MCAM | 2.72E-04 | 0.293125 | 0.055479 | 0.006711 |
| 34 | GBA | 1.01E-04 | 0.250625 | 0.055527 | 0 |
| 35 | GALNT13 | 2.00E-04 | 0.276250 | 0.055544 | 0.006711 |
| 36 | BTD | 1.04E-04 | 0.258125 | 0.055550 | 0 |
| 37 | B3GNT2 | 5.41E-05 | 0.260000 | 0.055664 | 0 |
| 38 | CEMIP | 1.02E-03 | 0.300625 | 0.055724 | 0.006711 |
| 39 | QSOX2 | 1.91E-04 | 0.271875 | 0.055794 | 0.006711 |
| 40 | TFRC | 3.82E-04 | 0.286875 | 0.055830 | 0.006711 |
| 41 | FGFR1 | 3.98E-04 | 0.284375 | 0.055938 | 0.006711 |
| 42 | ANGPTL2 | 1.19E-04 | 0.239375 | 0.056028 | 0 |
| 43 | TRIL | 4.99E-04 | 0.281875 | 0.056093 | 0.006711 |
| 44 | CBLN1 | 1.15E-04 | 0.261875 | 0.056096 | 0 |
| 45 | SLITRK4 | 4.19E-04 | 0.280000 | 0.056174 | 0.006711 |
| 46 | METRN | 5.11E-05 | 0.234375 | 0.056189 | 0 |
| 47 | KIAA0319L | 5.10E-04 | 0.296875 | 0.056197 | 0.006711 |
| 48 | CA11 | 3.21E-04 | 0.273125 | 0.056226 | 0.006711 |
| 49 | CACNA2D2 | 6.66E-04 | 0.291250 | 0.056231 | 0.006711 |
| 50 | EXTL2 | 8.87E-05 | 0.269375 | 0.056236 | 0.006711 |
| **Up-regulated proteins** | | | | | |
| 1 | NEFM | 2.70E-12 | 0.917500 | 0.0328792 | 0 |
| 2 | SERPINA3 | 1.31E-05 | 0.767500 | 0.0514578 | 0 |
| 3 | CHI3L1 | 7.27E-05 | 0.751875 | 0.0556800 | 0 |

**Supplemental Table S3. Top 50 up- and down-regulated differential proteins with a q-value < 0.01 in PSP compared to PD plus HC.**

| **No.** | **Protein**  **name** | **P value** | **Mean of**  **bootstrap AUC** | **SD of**  **bootstrap AUC** | ***q*-value** |
| --- | --- | --- | --- | --- | --- |
| **Down-regulated proteins** | | | | | |
| 1 | ATP6AP2 | 2.55E-15 | 0.077813 | 0.026220 | 0 |
| 2 | EFEMP2 | 5.43E-12 | 0.107500 | 0.035735 | 0 |
| 3 | LAMP2 | 2.60E-11 | 0.156563 | 0.036763 | 0 |
| 4 | CHST12 | 8.79E-10 | 0.164688 | 0.040060 | 0 |
| 5 | NDRG4 | 9.75E-07 | 0.225938 | 0.042684 | 0 |
| 6 | B4GALT1 | 2.65E-08 | 0.194688 | 0.043020 | 0 |
| 7 | MINPP1 | 4.12E-06 | 0.245938 | 0.043677 | 0 |
| 8 | ADAM15 | 6.01E-07 | 0.236406 | 0.044476 | 0 |
| 9 | ST8SIA5 | 1.97E-05 | 0.260000 | 0.044826 | 0 |
| 10 | B3GNT2 | 1.24E-06 | 0.240625 | 0.044899 | 0 |
| 11 | ZP2 | 6.44E-07 | 0.228750 | 0.045102 | 0 |
| 12 | ANGPTL2 | 1.11E-06 | 0.228750 | 0.045546 | 0 |
| 13 | LCAT | 2.18E-07 | 0.201563 | 0.045723 | 0 |
| 14 | GGH | 5.41E-08 | 0.211250 | 0.045751 | 0 |
| 15 | TIMP3 | 1.29E-05 | 0.269063 | 0.045800 | 0 |
| 16 | VWA7 | 8.96E-05 | 0.306875 | 0.045816 | 0.004739 |
| 17 | CDH7 | 6.72E-06 | 0.244375 | 0.045911 | 0 |
| 18 | FBLN2 | 5.84E-07 | 0.225313 | 0.046093 | 0 |
| 19 | CRYM | 3.07E-06 | 0.252813 | 0.046266 | 0 |
| 20 | ATP6AP1 | 1.06E-07 | 0.210000 | 0.046286 | 0 |
| 21 | PCMT1 | 6.95E-07 | 0.221563 | 0.046315 | 0 |
| 22 | CLSTN3 | 1.27E-05 | 0.258438 | 0.046351 | 0 |
| 23 | PCSK1N | 2.31E-06 | 0.234375 | 0.046523 | 0 |
| 24 | LINGO3 | 9.27E-06 | 0.255000 | 0.046839 | 0 |
| 25 | BTD | 2.00E-05 | 0.277500 | 0.046961 | 0 |
| 26 | ENPP5 | 8.28E-06 | 0.250313 | 0.047023 | 0 |
| 27 | CHGB | 7.71E-06 | 0.244063 | 0.047061 | 0 |
| 28 | A4GALT | 1.28E-05 | 0.259375 | 0.047075 | 0 |
| 29 | CBLN3 | 5.19E-07 | 0.209063 | 0.047245 | 0 |
| 30 | SEMA3G | 1.11E-05 | 0.262813 | 0.047376 | 0 |
| 31 | FAT2 | 1.49E-07 | 0.195000 | 0.047475 | 0 |
| 32 | METRN | 8.50E-06 | 0.248438 | 0.047573 | 0 |
| 33 | GRIA2 | 1.38E-04 | 0.288125 | 0.047601 | 0.004739 |
| 34 | FSTL5 | 8.41E-07 | 0.211875 | 0.047642 | 0 |
| 35 | SLITRK2 | 1.38E-05 | 0.261875 | 0.047919 | 0 |
| 36 | GRIA4 | 1.26E-05 | 0.250313 | 0.048000 | 0 |
| 37 | SERPINE2 | 1.15E-04 | 0.287500 | 0.048055 | 0.004739 |
| 38 | KCTD12 | 2.96E-06 | 0.245781 | 0.048059 | 0 |
| 39 | QSOX2 | 3.17E-05 | 0.258125 | 0.048067 | 0 |
| 40 | PREP | 6.19E-05 | 0.268750 | 0.048157 | 0 |
| 41 | MGAT5 | 4.40E-06 | 0.245313 | 0.048285 | 0 |
| 42 | MFGE8 | 5.38E-05 | 0.274375 | 0.048325 | 0 |
| 43 | SLITRK4 | 7.12E-05 | 0.279688 | 0.048350 | 0 |
| 44 | GBA | 1.66E-05 | 0.269375 | 0.048432 | 0 |
| 45 | CAMK2B | 1.61E-04 | 0.296875 | 0.048504 | 0.004739 |
| 46 | TIMP4 | 1.55E-05 | 0.254063 | 0.048568 | 0 |
| 47 | CHST10 | 7.36E-06 | 0.251250 | 0.048626 | 0 |
| 48 | BDNF | 4.72E-05 | 0.276250 | 0.048647 | 0 |
| 49 | DPP7 | 6.77E-04 | 0.312500 | 0.048654 | 0.004739 |
| 50 | PDYN | 3.22E-05 | 0.272813 | 0.048694 | 0 |
| **Up-regulated proteins** | | | | | |
| 1 | NEFM | 5.25E-12 | 0.89250 | 0.0320251 | 0 |
| 2 | SERPINA3 | 1.79E-06 | 0.76375 | 0.0437682 | 0 |
| 3 | CHI3L1 | 7.80E-06 | 0.75250 | 0.0438022 | 0 |

**Supplemental Table S4. Overlapping differentially expressed proteins between PSP GP and PSP CSF.**

| PSP vs HC | PSP vs PD | PSP vs PD plus HC |
| --- | --- | --- |
|  |  | ATP1B2 |
| CNTNAP2 |  | CNTNAP2 |
| EPDR1 |  | EPDR1 |
|  |  | FBLN2 |
| GGH | GGH | GGH |
|  |  | GOT1 |
|  | HAPLN4 | HAPLN4 |
|  |  | PREP |
| SERPINE2 |  |  |


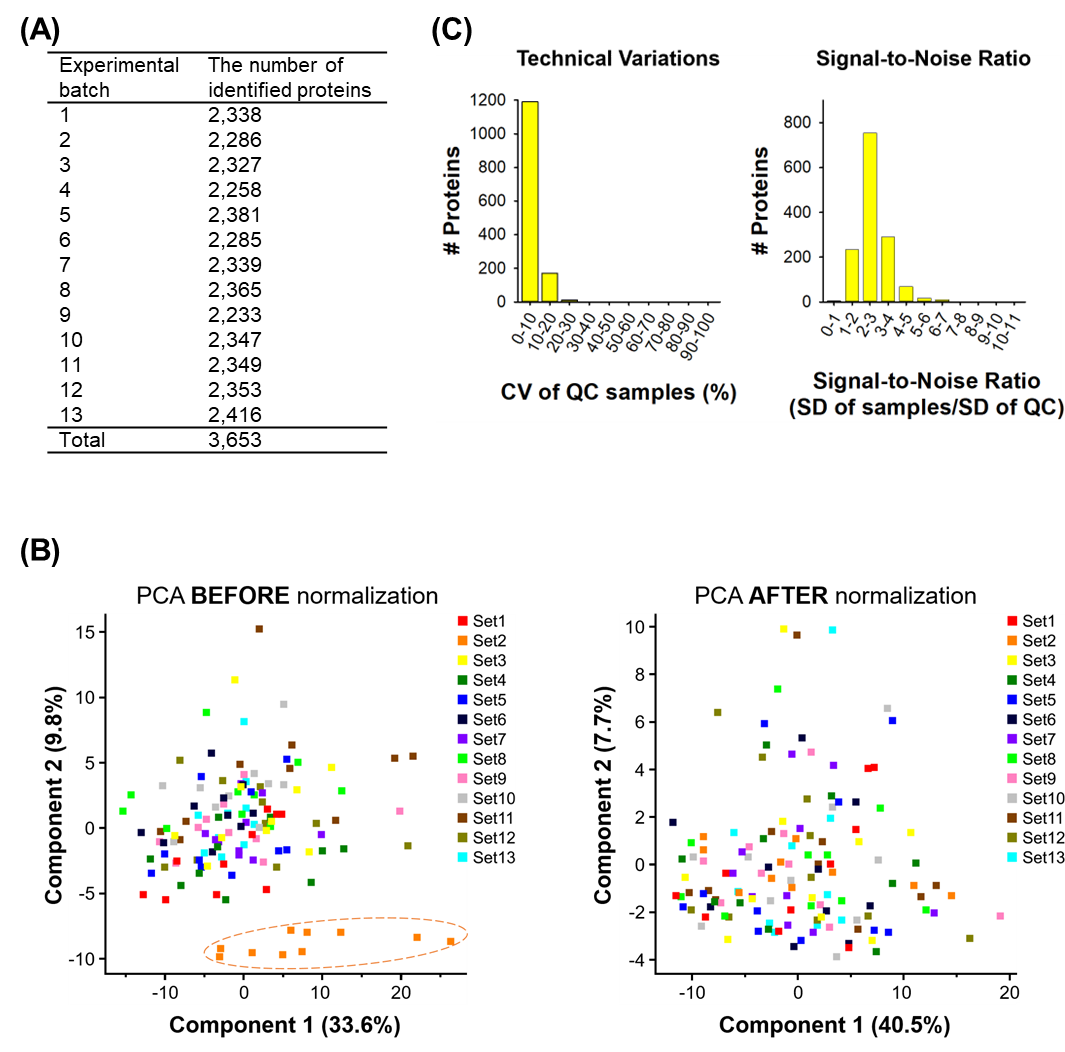


**Supplemental Figure S1. The evaluation of proteomic data quality from 13 batches of 11-plex TMT experiments.**

(A) The number of identified proteins in each batch. (B) To minimize batch effects of 13 different 11-plex TMT experiments, they were further normalized using the Combat package after normalizing each batch using MP. One hundred twenty CSF samples were shown on a 2D PCA plot to visualize potential batch effects before (left panel) and after (right panel) the normalization using the Combat package. (C) The coefficient of variation (CV) of QC samples and signal-to-noise ratio were calculated to evaluate data quality.


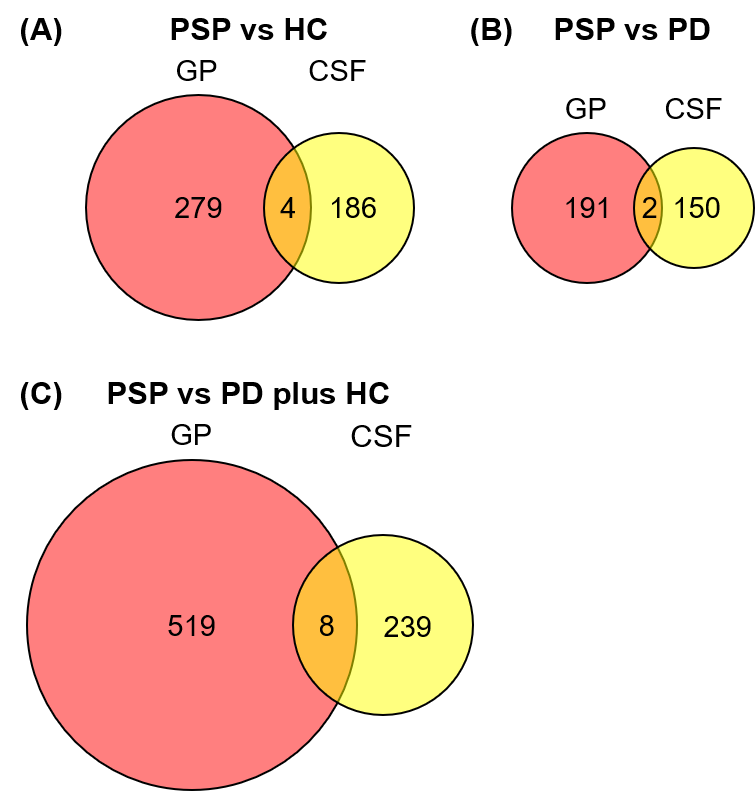


**Supplemental Figure S2. Comparison of differentially expressed proteins in CSF of PSP with the ones in GP of PSP.**

The differentially expressed proteins in CSF of PSP discovered in this study were compared with the ones in GP of PSP discovered in the previous study.^17^ We used *q*-values < 0.01 for the cutoff to determine differentially expressed proteins.


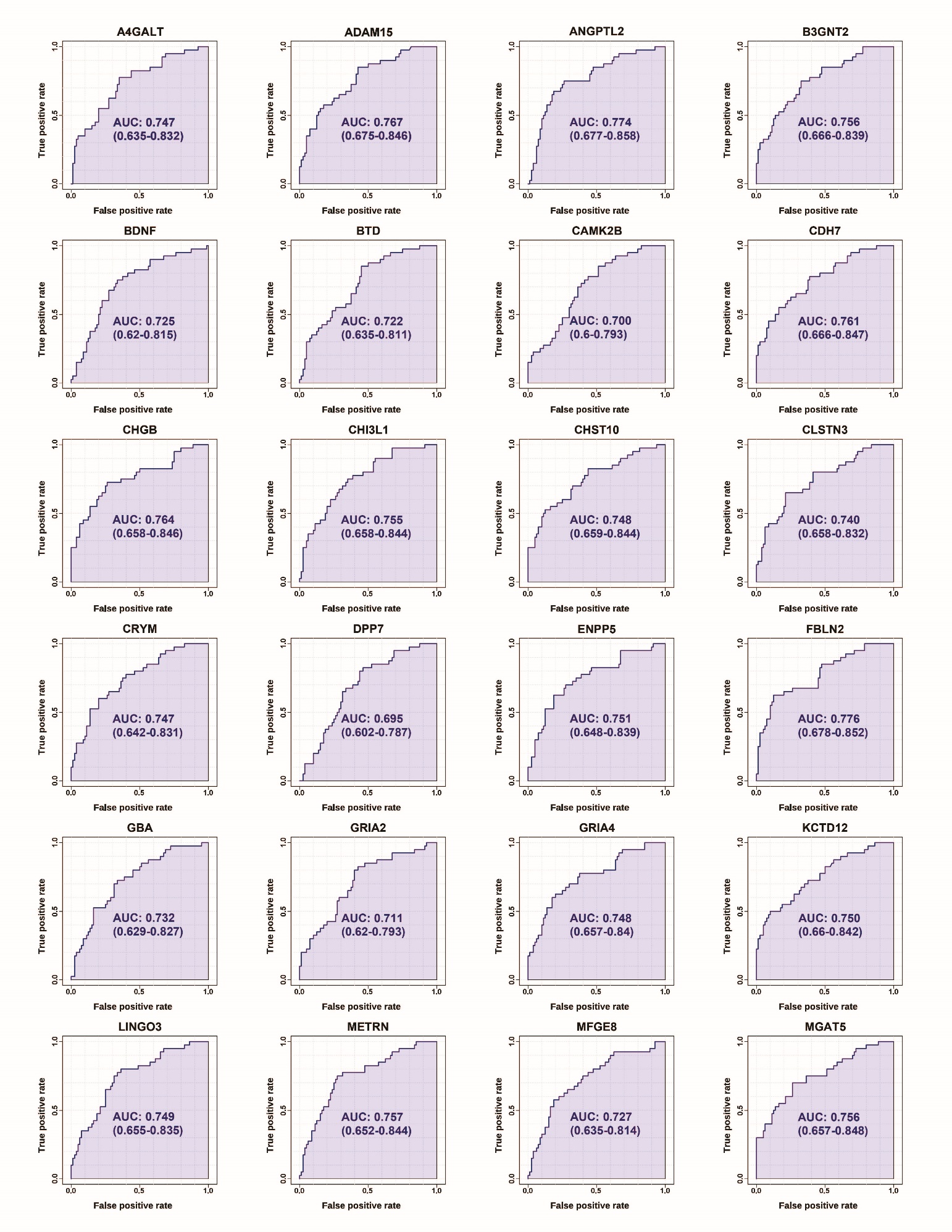


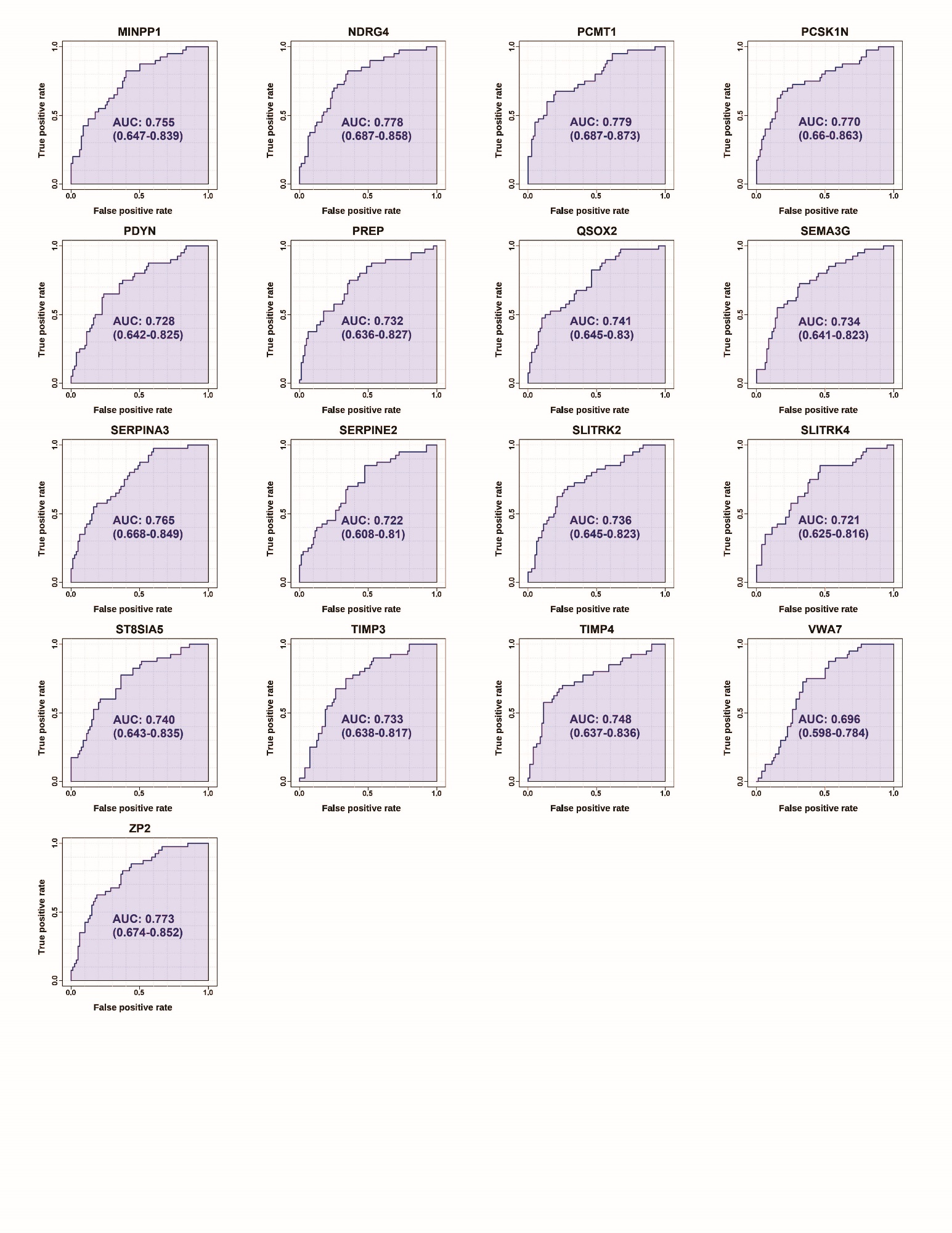


**Supplemental Figure S3. ROC analyses for the candidate PSP biomarker proteins.**

The discriminating capabilities of candidate PSP biomarkers were estimated by comparing PSP to PD plus HC using ROC analysis. ROC curves were generated by bootstrapping. The values in the parenthesis show the lower and upper AUC values of a 95% confidence interval. The X-axis denotes a false positive rate (1-specificity), and the Y-axis denotes a true positive rate (sensitivity).


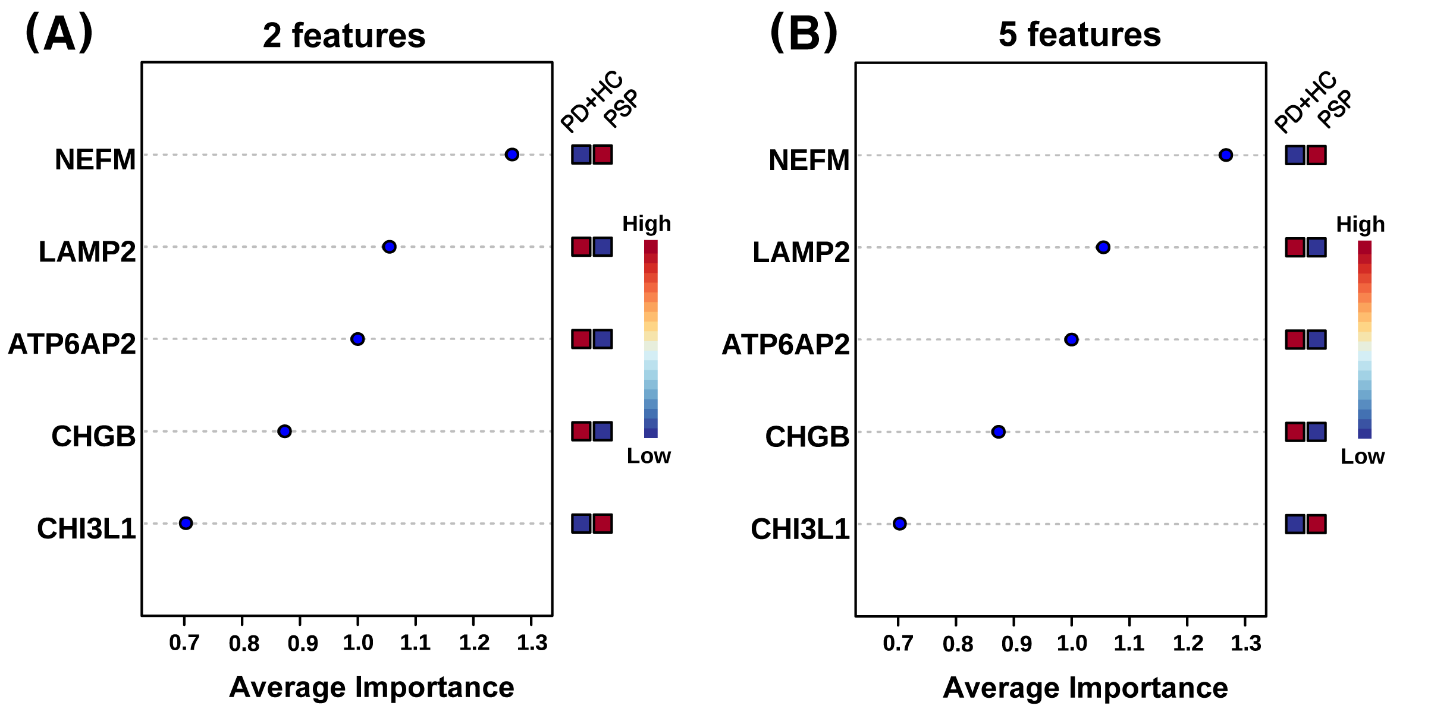


**Supplemental Figure S4. Average importance of features of the multivariate analyses using the top 5 features**

(A) A multivariate analysis to discriminate PSP from PD and HC was conducted with 2 out of the top 5 features. (B) A multivariate analysis to discriminate PSP from PD and HC was conducted with 5 out of the top 5 features.
